# Fate-tracking early coral recruits following bleaching in a remote reef ecosystem

**DOI:** 10.1007/s00338-025-02732-8

**Published:** 2025-09-03

**Authors:** John E. Stratford, Andrew O. M. Mogg, Heather J. Koldewey, Liam Lachs, Renata Ferrari, James Guest, Daniel T. I. Bayley

**Affiliations:** 1 Centre for Biodiversity and Environment Research, University College London, Bloomsbury, London, WC1H 0AG UK; 2 School of Natural and Environmental Sciences, Newcastle University, Newcastle Upon Tyne, UK; 3 Australian Institute of Marine Science, Townsville, QLD 4810 Australia; 4 Dunstaffnage Marine Laboratories, Tritonia Scientific Ltd., Oban, UK; 5 Zoological Society of London, Regent’s Park, London, UK; 6 Centre for Ecology and Conservation, University of Exeter, Penryn Campus, Cornwall, UK

**Keywords:** Reef recovery, Coral bleaching, Coral recruits, Chagos Archipelago, Photogrammetry

## Abstract

As coral reefs face increasingly frequent and severe disturbances, their condition relies more heavily on recovery dynamics. Understanding reef recovery is essential for assessing the long-term ecological integrity and functioning of these ecosystems. In this study, we used structure-from-motion photogrammetry to map reefs at Peros Banhos atoll (Chagos Archipelago) in the three years following the 2015–2016 mass coral bleaching event. This approach enabled us to detect and track individual post-bleaching coral recruits underpinning natural recovery (*n* = 1,074 across 72 m^2^), and investigate their early survival and growth. In 2017, one year after the bleaching, new recruit density was highest, largely due to comparatively high recruitment in sheltered sites. However, 2018 recruits had higher first-year survival and growth than the 2017 cohort, suggesting a negative legacy effect of high temperatures on reef recovery. Branching coral taxa showed both the highest first-year survival and growth. Interestingly, fine-scale substrate complexity at the onset of recovery was negatively associated with the density of recruits 1–2 years later. Despite favourable conditions that allowed the majority of recruits to survive and grow rapidly, all recruits combined accounted for only 2.39% coral cover three years after the bleaching event. Our results document vital rates during early natural recovery at a remote protected atoll and shed light on the dynamics of coral recruits immediately following mass bleaching. Further, we demonstrate the insight that photogrammetric approaches can provide to reef demographic studies.

**Supplementary Information:**

The online version contains supplementary material available at 10.1007/s00338-025-02732-8.

## Introduction

Acute disturbances play a critical role in structuring communities in high diversity ecosystems such as coral reefs (Connell 1978). There have been substantial global declines in coral cover over recent decades (Souter et al. 2021; Tebbett et al. 2023), and the frequency of loss due to acute disturbances is accelerating (Hughes et al. 2017; Hughes, Kerry, et al. 2017). The marine heatwaves and storms responsible for much coral cover decline are expected to become more frequent and severe over the coming decades against the backdrop of continued ocean warming, shortening the intervals between disturbance events (Cheal et al. 2017; Hughes et al. 2018; Intergovernmental Panel on Climate Change (IPCC), 2023). As such, reef recovery immediately following disturbances (such as mass bleaching and storm damage) is becoming an increasingly influential factor in the maintenance of ecological integrity and functioning of reefs (Montoya 2021). Understanding early-stage reef recovery dynamics is vital to maximising the efficacy of reef conservation, for instance, by identifying reefs with different recovery potential or by providing benchmarks against which to assess active restoration efforts (Beyer et al. 2018; Ferrari et al. 2021; Gann et al. 2019; Guest et al. 2018).

Following high mortality where few surviving colonies remain, the recruitment, growth and survival of new coral colonies are key drivers of reef recovery, such that recruit demography can be crucial to understanding early recovery dynamics (Edmunds & Riegl 2020; Kayal et al. 2018). While the fates of colonies that survive a disturbance also influence recovery, the focus of this study is on colonies that are first observed after the bleaching event (i.e. ‘post-bleaching recruits’) on reef patches where there were very few surviving colonies. Recruit demography is dependent on multiple interacting local conditions; for instance, time since disturbance, local wave exposure regimes, benthic composition and structure, temperature and latitude have all been found to influence aspects of recruit abundance, growth or survival (Carlson et al. 2024; Gilmour et al. 2013; Holbrook et al. 2018; Lange et al. 2021; Morais et al. 2024; Nozawa et al. 2021; Tebbett et al. 2022; Yanovski & Abelson 2019). Whilst previous research has established that each of the individual vital rates contributing to recovery dynamics are sensitive to local conditions, few studies have been able to assess all three rates concurrently, limiting understanding of their interactions and the overall system. What’s more, recruitment dynamics specifically following bleaching events can be influenced by further factors still. Following high bleaching-induced mortality, larval supply can remain minimal for several years in cases where few reproductive colonies remain and inter-reef connectivity is low (Gilmour et al. 2013). And elevated temperatures can have downstream physiological effects on surviving colonies, enabling bleaching events to have ecological legacy effects that extend beyond immediate mortality (Bouwmeester et al. 2023; Cantin & Lough 2014; Gold & Palumbi 2018; Hazraty-Kari et al. 2022; Leinbach et al., 2021). Such legacy effects may influence the earliest recruits emerging after bleaching, potentially introducing further variability and having implications for recovery trajectories. Improved understanding of recruit demography requires the longitudinal tracking of recruits through time, but the logistical challenges of tracking large cohorts of small recruits have historically constrained such research.

Over the last decade, developments in producing high fidelity three-dimensional reconstructions of reefs via the process of photogrammetry have provided novel opportunities for studying reef processes (Bayley & Mogg 2020; Burns et al. 2015; Lange & Perry 2020; Remmers et al. 2024a, b; Young et al. 2017). Via photogrammetry, many overlapping photographs are stitched together to provide scaled digital reconstructions from which measurements of any depicted features can be extracted, ex situ. This approach shifts labour- and time-intensive tasks (e.g. relocating and measuring individual colonies) from the field to the computer screen, facilitating the study of larger sample sizes across broader spatial scales than previously feasible under the logistical constraints of marine fieldwork. Photogrammetry has already been successfully employed to accomplish various reef analyses, including accurately measuring individual coral colonies (Ferrari et al. 2017; Lachs et al. 2023; Lange et al. 2022), quantifying reef surface complexity (Bayley et al. 2019; Fukunaga et al. 2020; Pascoe et al. 2021; Torres-Pulliza et al. 2020), studying reef community composition, even at submillimetre scales (Bayley & Mogg 2023; Gouezo et al. 2023; Hernández-Agreda et al. 2024) and tracking recruit survival (Sarribouette et al. 2022). Notably, photogrammetric methods have particular value in quantifying small changes that unfold over extended time periods, making them well suited to tracking the growth and survival of individual coral colonies (Ferrari et al. 2021). What’s more, photogrammetry enables straightforward quantification of benthic structural complexity—a feature known to significantly influence recruitment dynamics (Carlson et al. 2024; Graham & Nash 2013; Hata et al. 2017; Randall et al. 2021; Yanovski & Abelson 2019). This approach presents valuable opportunities for novel investigation of not only recruitment dynamics, but also their relationship with underlying structural complexity.

**Fig. 1:**
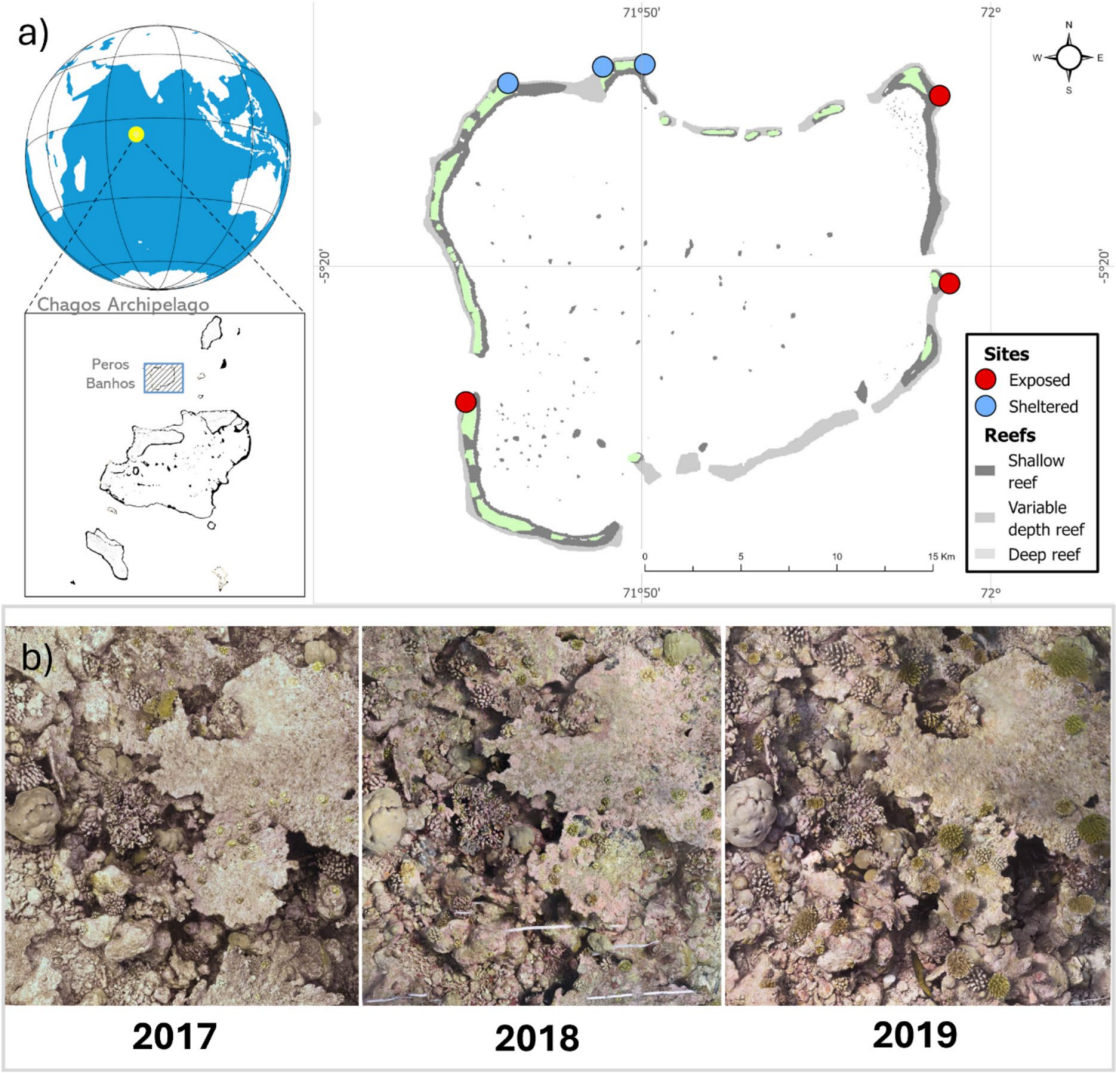
**a** The locations and exposure categories of the six sites surveyed around Peros Banhos atoll, northern Chagos Archipelago, in April 2017, 2018 and 2019. ‘Sheltered’ and ‘Exposed’ sites experienced wave exposure of 100–500 J m^−3^ and 800–1400 J m^−3^, respectively, using site exposure regimes modelled by Perry et al. (2015). **b** Time-series of orthomosaics of one of 18 quadrats imaged across each of the three years following mass bleaching

Situated in the centre of a large ‘no-take’ Marine Protected Area (MPA), 1,600 km from the nearest continent, the reefs of the Chagos Archipelago (Central Indian Ocean, Fig. 1) are some of the most remote in the world and experience only limited exposure to local anthropogenic stressors (e.g. overfishing and pollution from terrestrial waste (Sheppard et al. 2012)). As a result, reefs here offer a rare opportunity to study ecological processes without the interactions of multiple localised stressors, helping to clarify underlying trends that may be obscured in more impacted environments. Despite its protection and isolation, the Chagos Archipelago was exposed to the 2015–2016 prolonged marine heatwave which caused the most severe bleaching event on record, encompassing 51% of the world’s coral reefs (Eakin et al. 2018). This event caused the loss of up to 98% of coral cover in the worst-affected areas globally (Eakin et al. 2018). In the Chagos Archipelago, it led to the loss of 60% coral cover, with losses reaching 80% on certain reefs (Hays et al. 2020; Head et al. 2019). In this study, we utilise the years immediately following this mass mortality to study early natural reef recovery dynamics in an environment where few surviving colonies remain and investigate this process in the absence of potentially conflating localised stressors. We used photogrammetry to construct detailed large-scale reconstructions of six reef sites at an atoll in the Chagos Archipelago from 2017 to 2019, enabling us to (a) track a large number of post-bleaching recruits across the first three years following bleaching (*n* = 1,074) and (b) easily quantify local habitat complexity. To investigate early recruit demography, we quantified the recruitment (recruit density) and subsequent first-year survival and growth in both the first and second annual cohorts of post-bleaching recruits. We also determined the association between these vital rates and wave exposure, a factor known to strongly structure reef communities (Morais et al. 2024; Perry et al. 2015; Tebbett et al. 2022). Finally, we investigated the relationship between recruit vital rates and reef surface complexity in 2017 to explore whether surveys of post-disturbance benthic complexity could be used to predict variation in early recovery dynamics.

## Methods

### Site location

Six study sites were selected on seaward sloping reefs around the Peros Banhos atoll in the north of the Chagos Archipelago (Central Indian Ocean; Fig. 1). Peros Banhos’ reefs suffered extensive coral mortality in the 2016 mass bleaching event (Hays et al. 2020; Head et al. 2019). All sites were surveyed at permanently marked locations at depths of 8–12 m in April 2017, 2018, and 2019, spanning the three years immediately following the high mortality observed in the 2016 bleaching event (Head et al. 2019).

To assess recruit demography at reefs under different wave exposure regimes, we selected three sites as ‘Sheltered’ sites (exposure within 100–500 J m^−3^), and three as ‘Exposed’ sites (exposure within 800–1400 J m^−3^; Fig. 1). Exposure regimes of these six sites (149–1,398 J m^−3^) were estimated in Perry et al. (2015), where spatially explicit estimations of wave exposure were modelled as a function of wind speed and direction and fetch length (see Perry et al. (2015) for further details).

### 3D photogrammetry survey

We used ‘Structure from Motion’ (SfM) photogrammetry to create Digital Elevation Models (DEMs) and large-scale ortho-rectified photomosaics (i.e. high-definition photo maps; referred to as ‘orthomosaics’ hereafter) of each site, following the procedure detailed in Bayley & Mogg (2020). In brief, approximately 100 m^2^ (10 m by 10 m) of reef flat at 8–12 m depth was photo-surveyed at each site by taking sequential overlapping photos (75–80% overlap) of the benthos via a ‘lawn-mower’ survey pattern. Photos were taken approximately 0.5–1 m directly above the benthos, using a single Digital Single-Lens Reflex (DSLR) camera under natural lighting (see Table S1 for camera specifications and settings). Ground Control Points (GCPs) of known dimensions and a spirit level were placed within each site at known depths and included in the photo-survey to enable scaling and consistent top-down orientation of the 3D reconstructions. Surveys were carried out in April 2017, 2018 and 2019, providing images of the same reef patches in the three consecutive years following bleaching. To ensure re-sampling of the same areas across the three years, a permanent GPS-marked steel stake was deployed in each site, along with three smaller steel markers at each other corner of the 10 m × 10 m plots. Plot boundaries were measured and delineated with in situ tapes, and surveys deliberately included a buffer zone of 1 m past the tape to ensure full image capture of the core area, GCPs, and spirit level. We mapped the larger 100 m^2^ sites rather than individually mapping the 4 m^2^ quadrats that were ultimately used to allow faster survey of all quadrat locations, to include area for further replicates as contingency, and because the data was also intended for use in additional studies (e.g. Bayley & Mogg (2023)).

Agisoft Metashape Pro (v1.6.3; Agisoft, St. Petersburg, Russia; https://www.agisoft.com/) was used to build 3D reconstructions of each site in each study year, using the settings detailed in Table S1. A DEM and an orthomosaic (resolution = 0.57 ± 0.12 mm pixel^−1^ and scale bar error = 0.0046 ± 0.0026 m (mean ± SD)) were generated from each 3D reconstruction and used for our analyses of reef communities. Each were orientated from a bird’s-eye view (i.e. perpendicular to the vertical *Z* axis), as adjudged using the spirit-level.

### Analyses of coral recruits

To quantify recruit density, growth and survival, three virtual 2 m × 2 m quadrats were outlined on the orthomosaic of each site in 2017 using the ‘Draw polygon’ tool within Metashape (v1.6.3). To investigate the recovery of areas where there were few surviving corals, and to minimise the likelihood of missing recruits hidden beneath large overhangs, quadrats were intentionally drawn on flat areas dominated by large dead table coral or bare bedrock in 2017. Quadrats were then redrawn in the same location in the corresponding orthomosaics of 2018 and 2019. This was achieved by manually locating features (e.g. coral bommies or distinctive colonies) present across 2017, 2018 and 2019 orthomosaics of each site and placing quadrats in relation to these references. To enable standardisation for the total area in each quadrat available for potential colonisation by coral recruits, we traced and measured (within Metashape) the total planar area of substrate unsuitable for coral recruits (such as surviving live coral, sand, and small loose rubble) within each quadrat in 2017 and then subtracted this area from the total planar quadrat area.

We investigated the density, survival and growth of all colonies that we deemed to be post-bleaching recruits that were visible within each quadrat between 2017 and 2019. Here, we use the general form of the term ‘recruit’, recognising all colonies that arrive between surveys as recruits, following a principle commonly used in recruitment monitoring (Edmunds 2023). Strictly, recruits are defined here as all colonies believed to have reached a size visible to the naked eye within a high-resolution orthomosaic within the preceding year (i.e. within the survey interval period). The smallest recruit we detected was 0.2 cm^2^, equivalent to a circle of 0.55 cm diameter (mean: 6.0 cm^2^ or 2.8 cm diameter; Fig. S1). Some recruits smaller than 0.2 cm^2^ were likely present but not detected, but this limitation was consistent across the whole dataset since the same methods were used throughout. Recruits located under overhangs or within crevasses were likely not visible due to the two-dimensional nature of orthomosaics, however intentional placement of quadrats on largely flat areas (e.g. atop dead tabular *Acropora*) minimised this limitation. We also acknowledge that a small number of recruits may have been missed when first present, or recorded as new recruits when they were in fact older (discussed further below). We located and measured the planar area of all juvenile coral colonies visible within 2017 survey quadrats and measured them again (or noted their absence) in the corresponding 2018 and 2019 quadrats. The planar surface area of each colony in each year was measured within Metashape by tracing the colony perimeter on the scaled orthomosaics and extracting the area of the resulting polygon. Where colonies were blurred such that their boundaries were unclear, their area was not recorded. Colonies with an overgrowing neighbour colony or which grew on an incline, were recorded based on the area visible from directly above (as consistently viewed in orthomosaics across all years). We then added any new recruits that became visible in 2018 or 2019 in the same way, such that all recruits believed to have settled within three years of bleaching (April 2016–2019) within each quadrat were located and measured each year that they were present.

With no data prior to 2017 it is not possible to be certain whether colonies observed in 2017 are new, post-bleaching recruits (i.e. whether they reached a visible size within the preceding year) or are older survivors of the bleaching, and it is possible that the cohort of colonies termed ‘2017 recruits’ includes some colonies that were older than one year old during 2017 surveys. The following measures minimised this as much as is possible: Firstly, the majority of colonies included in this study were found on coral skeletons of tabular *Acropora* colonies that likely died in the 2016 bleaching event. Secondly, after having become proficient at identifying recruits in the 2018 and 2019 orthomosaics (where their absence in the previous year could be verified), a single observer (JS) critically re-inspected the size and morphology of all colonies present in 2017. Colonies present in 2017 that were notably more structurally developed than the verified recruits observed in 2018 and 2019 were excluded from the study, and the remaining colonies were termed ‘2017 recruits’. The mean colony size of 2017 recruits was within the range of verified recruits observed in subsequent years (2017: 6.0 cm^2^ or 2.8 cm diameter; 2018: 5.9 cm^2^ or 2.7 cm; 2019: 6.4 cm^2^ or 2.9 cm), suggesting that the inclusion of colonies older than one year old in 2017 was minimised (Fig. S1). However, there is evidence that some recruits have capacity to persist at small size classes (potentially below visual detection size) and then later grow to take advantage of newly available space when given opportunity (Doropoulos et al. 2022), such that some colonies may have settled earlier than the size suggests. As a study investigating recruitment to natural substrate (rather than to artificial substrate known to be free of pre-existing coral spat), there is some inevitable uncertainty regarding when recruits settled.

Recruit density was calculated annually as the number of new recruits observed in each year (i.e. for 2018 and 2019: all recruits not observed in previous years; for 2017: all colonies observed in 2017 that matched the size range and morphology of confirmed recruits from 2018 and 2019) per m^2^ of suitable substrate. 2017 and 2018 recruits were adjudged to have survived their first year of life if they were visible and alive in 2018 and 2019, respectively. If a colony died or disappeared during the study, we noted the likely reason for mortality by inspecting its locality both when it was last observed and first absent. We categorised colony mortality or disappearance as due to either substrate collapse, competition/overgrowth by neighbouring colonies, total colony disappearance (e.g. through scouring, predation or wave action) or dead in situ (with the skeleton remaining visible). No statistical analyses were conducted regarding the circumstances of colony loss because only 27 2018 recruits died or disappeared. Annual growth (cm^2^ year^−1^) was calculated as the change in a colony’s planar area between consecutive years. Since our goal was to assess how suitable the environments were for recruit growth, we excluded the 43 colonies that shrank between years from growth analyses (removing 6.5% of colonies measured in consecutive years). Reduction in colony size is driven by different factors than positive growth (e.g. corallivory, abrasion) so exclusion of these colonies enabled more accurate assessment of physiological growth rates—though colonies that displayed positive growth may still have experienced partial reductions due to corallivory and abrasion. Recruit coral cover of each quadrat was calculated as the sum of the planar area of all post-bleaching recruits and standardised for the total area of each quadrat.

All recruits were identified to genus level where possible, or otherwise to family level or classed as unidentifiable. Where taxonomy could not be inferred from the orthomosaics, we consulted the underlying photographs for higher quality images. We also recorded the associated growth form of each colony from visual inspection of colonies at their most matured state during the study period, classifying each colony as either tabular, foliose, digitate, corymbose, encrusting, massive, or sub-massive (or ‘unidentified’ where colonies remained without a clear growth form). Each recruit was then also assigned to one of five morphotaxa categories (where the categories used were informed by the number of each genus and growth form combination observed): *Acropora*, other branching, encrusting, massive, or unidentified.

### Surface complexity in 2017

To enable investigation of if benthic topological complexity shortly after bleaching-induced mortality was a predictor of subsequent recovery dynamics at Peros Banhos, we quantified the surface complexity of each quadrat in 2017. Considering that quadrats were intentionally placed on generally flat areas (e.g. areas dominated by dead tabular *Acropora* and reef pavement), here we look at the effect of complexity in areas where it is relatively low. Following the methodology reported in Fukunaga et al. (2019), we quantify benthic complexity using Digital Elevation Models (DEMs). DEMs were exported from Metashape at 1 cm raster resolution (comfortably within our 1.3–5.1 mm range of model accuracy (Control scale bars Total Error) and in accordance with Fukunaga & Burns (2020)) then analysed in R following Fukunaga et al. (2019). For each quadrat we calculated two habitat metrics: surface complexity (at 30 cm resolution) and fractal dimension (across 1, 2, 4, 8, 16 and 32 cm resolution). Surface complexity (SC) reports the ratio between planar area and observed surface area and is analogous to the chain-and-tape surface rugosity measure traditionally used in reef ecology (Young et al. 2017). SC at 32 cm resolution investigates complexity provided by medium-scale benthic features such as large rubble fragments and dead coral colonies. Calculation at much lower-resolution would investigate complexity provided by large reef features (e.g. bommies and ridges), which is beyond the scope of this study and the extent of the 2 by 2 m quadrats used. We explored including SC at higher resolution (1 cm) but, consistent with Fukunaga et al. (2020), we found SC at 1 cm to be highly correlated with both SC at 30 cm and with fractal dimension (Pearson’s correlation coefficient, *r* = 0.82 and 0.96, respectively,* P* < 0.01) so excluded it from our analyses. Fractal dimension (FD) is a metric widely used in the quantification of reef complexity and is a measure of change in surface complexity across different scales (from 1 to 32 cm here), enabling investigation of the overall structural variability of a surface. FD was calculated as per Eq. (1), whereby *σ* is each resolution of the DEM and *S*(*σ*) is the observed surface area at the resolution *σ* (Young et al. 2017).

1 $${\text{Fractal}} \;{\text{Dimension }} = 2 - {\text{slope}}\; {\text{of }}\;\left[ {\log \left( {S\left( \sigma \right)} \right)/\log \left( \sigma \right)} \right]$$

FD of surfaces ranges between 2 and 3, where high FD values indicate that large proportions of a quadrat’s surface complexity is due to fine-scale complexity (such as small crevices and overhangs), and smaller values suggest that larger features contribute more.

### Statistical analyses

We used generalised linear mixed effect models (GLMMs) to investigate how recruit density and vital rates were influenced by their environment. Across six models, we tested associations between three predictors—years since bleaching, reef exposure category, and initial benthic fractal dimension—and three responses: (a) annual recruitment density, (b) first-year survival of recruits and (c) first-year growth of recruits.

The structure of all models is reported in full in Table S2. To model recruit density (Model 1) we included three predictors and an interaction as fixed effects: year, reef exposure category, and their interaction, and each quadrat’s fractal dimension in 2017. The unique quadrat ID was also included as a random effect. We used a similar approach when modelling first-year survival (Model 2) and growth (Model 3), but also included colony morphotaxa and initial colony surface area as fixed effects to account for taxa- and size-specific impacts on survival and growth. Throughout, surface complexity at 30 cm resolution (fixed effect) and site (random effect) were initially included but were ultimately excluded because their removal improved model fit, as assessed by AIC (Bolker et al. 2009). Recruitment, growth and survival were modelled using a Tweedie distribution under Log link, a Gamma distribution under log link, and a binomial distribution under logit link, respectively. Tukey tests were used to conduct post hoc pairwise comparisons of factors.

In addition to Models 1, 2, and 3, we developed Models 4, 5, and 6 to further explore specific interactions. Exploratory scatter plots indicated that the relationship between recruitment and initial fractal dimension varied by year. Therefore, we adapted Model 1 to create Model 4 by replacing the interaction between ‘recruitment year’ and ‘exposure’ with an interaction between ‘recruitment year’ and ‘fractal dimension’. To investigate the survival of different morphotaxa (Model 5), we employed a similar modelling approach and edited Model 2 to include the interaction term between ‘recruitment year’ and ‘morphotaxa’ (rather than ‘exposure’). To investigate morphotaxa-specific growth (Model 6) we edited Model 3 to include a three-way interaction between ‘recruitment year’, ‘exposure’ and ‘morphotaxa’.

All statistical analyses were conducted in R v.4.3.2 (R Core Team 2024), using the glmmTMB package (Brooks et al. 2017) to construct GLMMs and the emmeans package (Lenth et al. 2024) to conduct Tukey tests. Model validation was completed using visual assessment of residual plots, aided by the DHARMa R package (Hartig & Lohse 2022).

## Results

### Overall

A total area of 72 m^2^ of reef was searched and annotated in each of the three years (2017, 2018 and 2019), resulting in the recognition of 1,074 likely post-bleaching coral recruits from 18 different genera and 6 distinct colony growth forms.

Recruit density declined from 8.54 new recruits m^−2^ in 2017 (the first year after bleaching) to 2.88 new recruits m^−2^ by 2019 (the third-year post-bleaching; Table S3). Recruit first-year survival and median positive growth ranged from 45–100% and 4.1–20.0 cm^2^ yr^−1^, respectively (Table S4), varying significantly over time, exposure and taxa.

### Post-bleaching recruit density at Peros Banhos Atoll

**Fig. 2:**
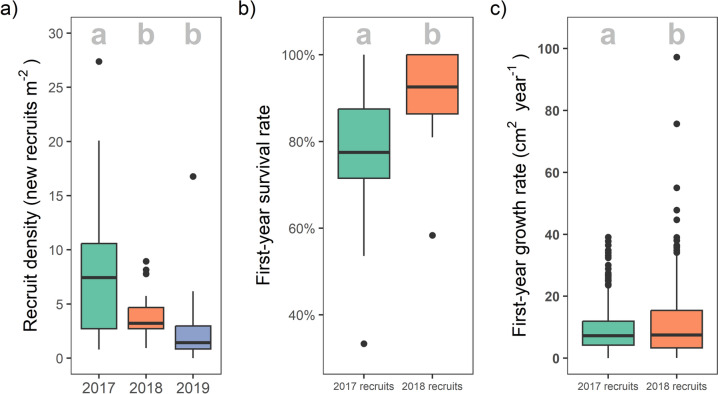
**a** Density, **b** first-year survival and **c** first-year growth of post-bleaching recruits in each year (showing the median, 25th and 75th percentiles, ± 1.5 IQR and outliers). Shared letters within plots denote groups with no statistically significant differences (*p* < 0.05)

We investigated the influence of wave exposure, fractal dimension and years since bleaching on the density of new recruits (Model 1 & 4). Pairwise Tukey tests from Model 1 revealed that the mean recruit density of all sites in 2017 (8.54 recruits m^−2^) was significantly higher than in both 2018 (4.05 recruits m^−2^;* Z* = 3.2, *P* = 0.004) and 2019 (2.88 recruits m^−2^; *Z* = 4.9,* P* < 0.0001). New recruit density was not statistically different between 2018 and 2019 (Z = 1.9,* P* = 0.14; Fig. 2a; Table S5).

**Fig. 3:**
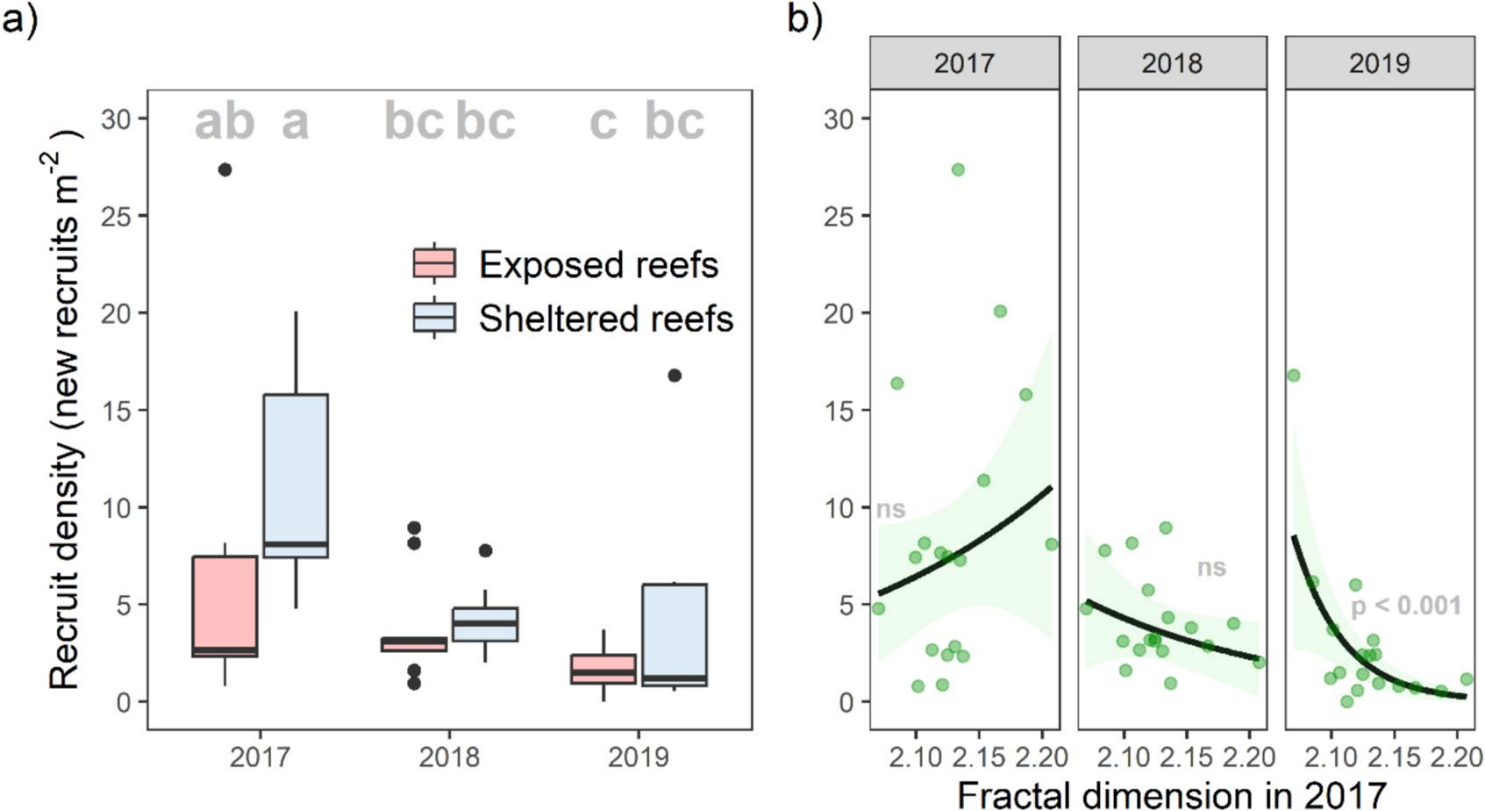
**a** Annual recruitment density in quadrats at exposed and sheltered sites in each of the three years following the 2016 mass bleaching (showing the median, 25th and 75th percentiles, ± 1.5 IQR and outliers). Shared letters denote groups with no statistically significant differences in recruitment density (*p* < 0.05). **b** The relationship between quadrat fractal dimension in 2017 and recruitment density in each year (showing the predictions of Model 4 (± SE), with the observed data overlaid). Inset text indicates the statistical significance of the relationship in each year (ns = not significant)

The effect of year on recruit density varied with wave exposure (Table S5, Fig. 3). On sheltered reefs, recruit density was significantly higher in 2017 than both 2018 and 2019. On exposed reefs, recruit density was significantly higher in 2017 than 2019 only. Within all years, there was no significant difference in recruit density between sheltered and exposed sites (Tukey, sig. level = 0.05, Table S5, Fig. 3).

The effect of initial fractal dimension on recruitment density varied between years (Model 4; Table S6). Fractal dimension in 2017 had a positive non-significant association with recruitment density in 2017, and a negative non-significant association in 2018. By 2019 fractal dimension in 2017 was significantly negatively associated with recruit density (Fig. 3b; emtrends,* Z* = −4.4, *P* < 0.001; Table S6).

### First-year survival of post-bleaching recruits

The influence of recruitment year, exposure and fractal dimension on the first-year survival rate of recruits was investigated in Model 2 and Model 5. In both models, initial colony size was significantly positively associated with survival (*p* < 0.05, Tables S7 and S8) and was accounted for in the models.

**Fig. 4:**
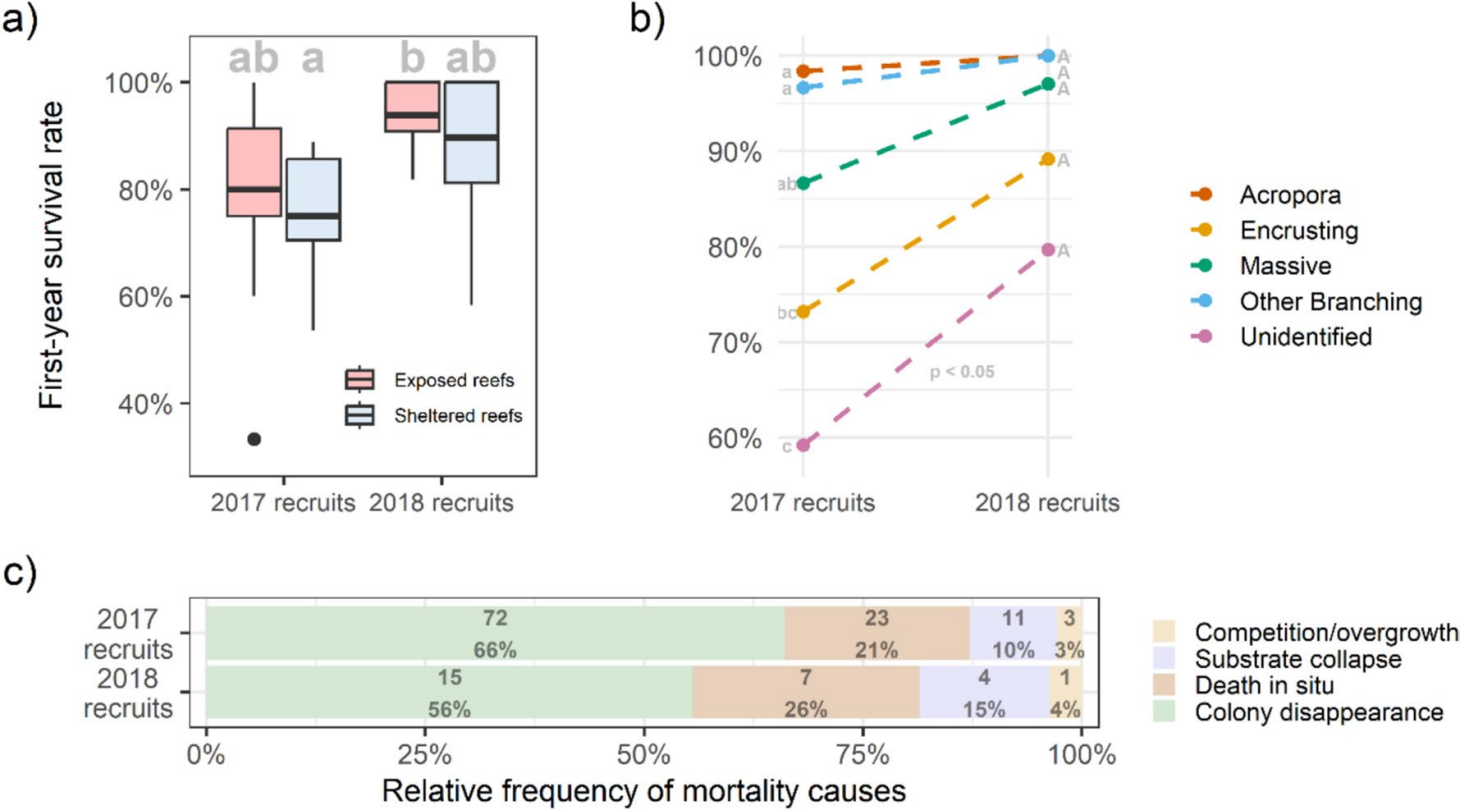
**a** First-year survival rates of 2017 and 2018 recruits observed in quadrats at exposed and sheltered sites (showing the median, 25th and 75th percentiles, ± 1.5 IQR and outliers). Shared letters denote groups with no statistically significant differences in growth rate (*p* < 0.05). **b** The first-year survival rates of the five morphotaxa groups in 2017 and 2018. Shared letters *within each year* denote groups with no statistically significant differences in survival rate (*p* < 0.05). Only unidentified colonies had significantly higher survival in 2018 than 2017. **c** The occurrence of circumstances of colony loss for all recruits that did not survive their first year of life (*n* = 109 among 2017 recruits and 27 among 2018 recruits), showing the raw number of colonies assigned to each category followed by the percentage contribution. The likely cause of mortality was inferred by inspecting a colony’s locality both when it was last observed and first absent. ‘Death in situ’ describes colonies for which the skeleton was observed with no live tissue, whilst all other categories apply to where the colony disappeared between years

Assessing the recruit assemblage as a whole (Model 2), first-year survival was significantly higher in 2018 (90.4% survival) than in 2017 (79.7% survival; Tukey,* Z* = − 2.9, *P* = 0.004; Fig. 2b; Table S7). Exposure had no significant effect on overall first-year survival in both 2017 and 2018 (Tukey, *P* > 0.4; Table S7; Fig. 4). Local initial fractal dimension was significantly negatively associated with colony first-year survival (Fig. 4; GLMM, *Z* = − 2.2,* P* = 0.028, Table S7).

Examining individual morphotaxa (Model 5) revealed that morphotaxa identity had a significant influence on first-year survival (Fig. 4). Considering survival within each morphotaxa, unidentified colonies (i.e. colonies that never developed a distinguishable growth form throughout the study period) had significantly greater first-year survival in 2018 than in 2017. No other morphotaxa had statistically different survival between 2017 and 2018. Between morphotaxa, in 2017 *Acropora* and ‘other branching’ colonies had significantly higher first-year survival than encrusting colonies. Massive colonies’ first-year survival was intermediary, with no significant difference between massive, *Acropora*, other branching or encrusting colonies. In 2018, no morphotaxa had significant differences in first-year survival (for statistics see Table S8).

In both 2017 and 2018 recruits, colony disappearance was the leading apparent cause of mortality within our sites, followed by death whilst remaining in situ, substrate collapse then overgrowth by nearby colonies (Fig. 4c).

### First-year growth of post-bleaching recruits

We measured the size of 660 colonies in two consecutive years. 617 colonies (93.5%) grew larger over the study period, whilst 43 colonies (6.5%) reduced in size (and were excluded from growth analyses, hence here we report on positive growth only).

We investigated the influence of recruitment year, exposure and fractal dimension on the first-year growth rate of recruits (Model 3 and Model 6). In both models, initial colony size was significantly positively associated with first-year growth (*p* < 0.005, Tables S9 and S10) and was accounted for in the models.

**Fig. 5:**
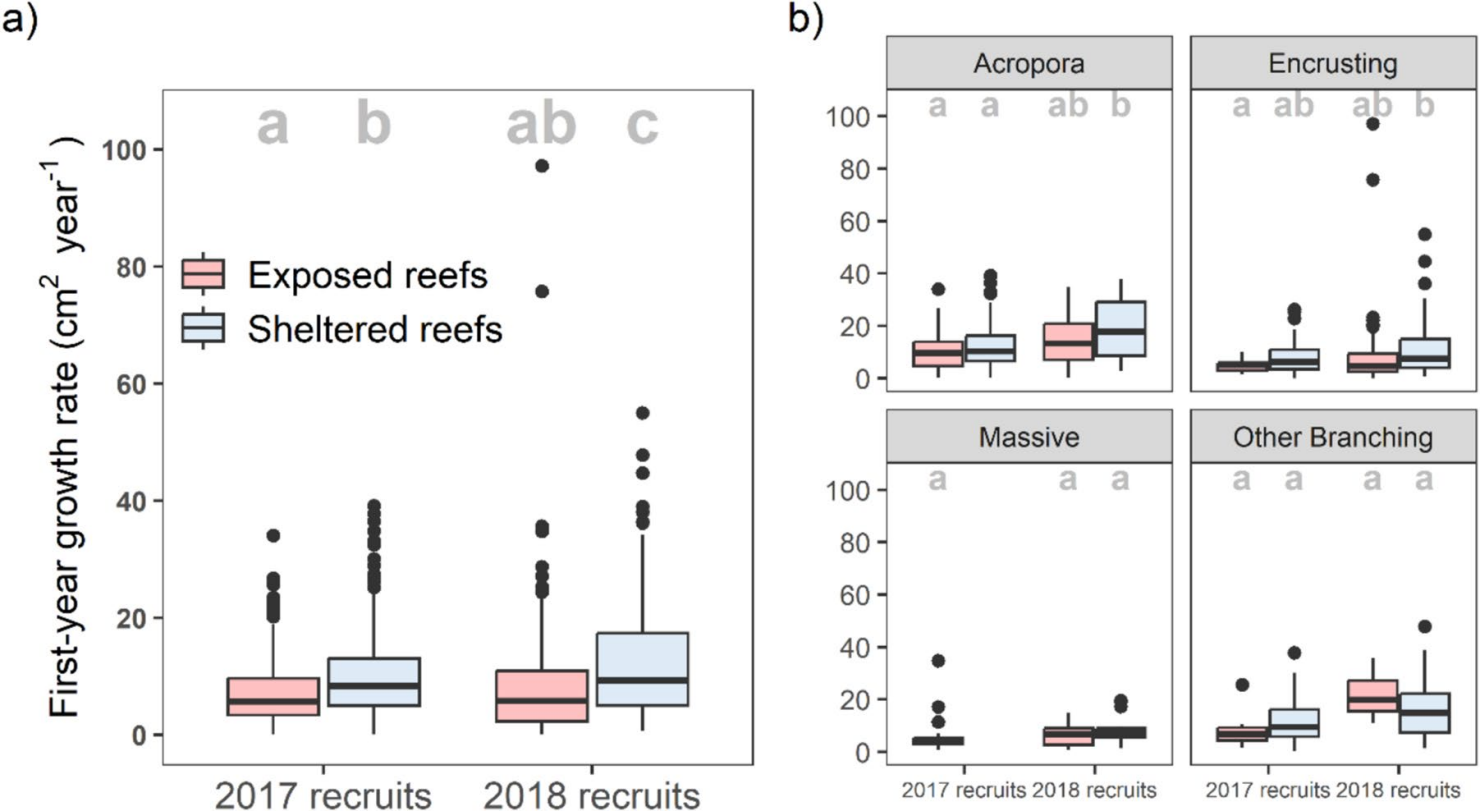
**a** First-year growth rates of 2017 and 2018 recruits at exposed and sheltered sites (showing the median, 25th and 75th percentiles, ± 1.5 IQR and outliers). Shared letters denote groups with no statistically significant differences in growth rate (*p* < 0.05). **b** First-year growth rates of four morphotaxa groups in 2017 and 2018. Shared letters within each morphotaxa denote groups with no statistically significant differences in growth rate (*p* < 0.05)

For the recruit assemblage as a whole (Model 3), first-year growth was significantly greater in 2018 recruits than in 2017 recruits (11.1 and 9.2 cm^2^ year^−1^, respectively; Tukey,* Z* = − 3.0, *P* = 0.002, Table S9; Fig. 2c; equivalent to 0.9 and 0.8 cm increases in colony radius, respectively). Specifically, growth rates were statistically consistent between years at exposed reefs (Tukey,* Z* = − 1.1, *P* = 0.689), and were only greater in 2018 than in 2017 at sheltered reefs (Tukey, *Z* =− 3.5, *P* = 0.003; Fig. 5, Table S9). In both 2017 and 2018 recruits, first-year growth was greater at sheltered reefs than at exposed reefs (Tukey, 2017: *Z* = − 2.6, *P* = 0.043; 2018: *Z* = − 4.0, *P* = 0.0004; Fig. 5). Initial fractal dimension was non-significantly negatively associated with the first-year growth of recruits, but with a high level of uncertainty (GLMM, *Z* = − 1.9*,* *P* = 0.056).

The first-year positive growth of recruits differed among morphotaxa (Model 6; Fig. 5). Both massive and other branching recruits had consistent growth across all year and exposure combinations. *Acropora* recruits had consistent growth within each year, but growth at sheltered sites in 2018 was significantly greater than growth at both sheltered and exposed sites in 2017. Encrusting recruits also had consistent growth within years, and had significantly higher growth at sheltered sites in 2018 than at exposed sites in 2017. Between morphotaxa, and across all sites and years, both *Acropora* and other branching recruits had significantly greater first-year growth than encrusting and unidentified recruits (for statistical results see Tables S10 and S11). Reporting of inter-morphotaxa statistical testing involving massive recruits was not possible due to the low number of massive recruits found.

### Post-bleaching recruit assemblage by 2019

**Fig. 6:**
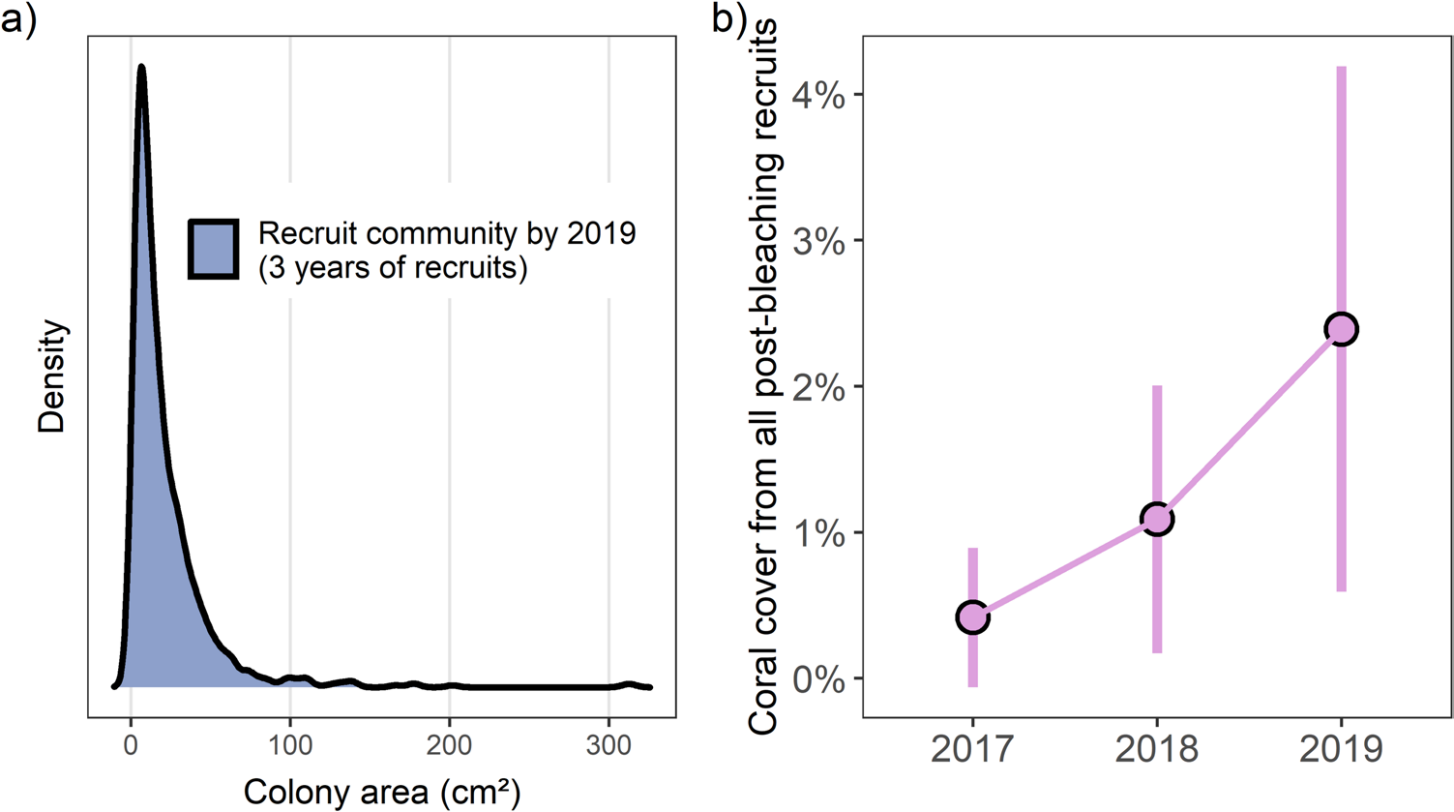
**a** The combined size frequency distribution of all colonies that were recruited after the bleaching (i.e. all 2017–2019 recruits). **b** Total coral cover provided by post-bleaching recruits (Mean ± SD) across the first three years following bleaching. Total coral cover at Peros Banhos atoll was 36% in 2012 (Head et al. 2019)

By 2019, mean (± SD) colony size across all three cohorts of post-bleaching recruits was 29.4 ± 38.9 cm^2^, equivalent to a circular colony of 6.12 cm diameter. The median colony size was only 13.4 cm^2^ (4.1 cm diameter) but two colonies—both *Acropora* recruited in 2017—exceeded 300 cm^2^ (19.5 cm diameter). Mean coral cover derived from post-bleaching recruits reached 2.39 ± 1.80% in 2019 (Fig. 6). Note that this percentage value applies to contributions from recruits only, and does not include cover provided by any large colonies that survived bleaching (however, such colonies were rare due to high post-bleaching mortality and our deliberate drawing of quadrats in areas with minimal surviving coral (Head et al. 2019)).

## Discussion

We fate-tracked 1,074 colonies over three years at Peros Banhos atoll, facilitating the compilation of an extensive dataset reporting density and subsequent survival and growth of post-bleaching recruits (Table S4). Whilst limited in its spatial and temporal extent, this constitutes one of few studies investigating all three of these demographic rates simultaneously, enhancing understanding of the early recruit population dynamics following bleaching-induced mortality at this remote atoll. The highest density of recruits was observed in the first year following bleaching (2017), but recruits in this cohort then grew less and were more likely to die in their first year after recruitment than the cohort that recruited the following year (2018). Surprisingly, greater local structural complexity was found to be associated with lower demographic rates in the recruits tracked here.

The process of reef recovery can take over 10 years and demography can vary drastically between reefs. Here we investigated recruit population dynamics in two cohorts of recruits at a single atoll between 2017 and 2019. Whilst limited in spatial and temporal extent, our results provide a detailed insight into what occurred on these reefs during this period and add to understanding of the variability of wider reef recovery. Future studies are required to investigate if the trends we observed here are acting at other locations and over longer time periods.

### Contextualising recruit demography at Peros Banhos

Different methods of quantifying recruitment (e.g. via imagery, settlement tiles or in-water juvenile surveys) can make comparison of recruit densities between different studies misleading so we do not directly compare the recruit densities reported here with those from other studies using different methods. However, our results corroborate other studies from this region showing that recruits have been abundant following bleaching events in the central Indian Ocean (Koester et al. 2021; McDevitt-Irwin et al. 2024; Sheppard et al. 2002). Generally, the recruits tracked here displayed high growth and survival rates. The annual growth of *Acropora* recruits that we observed (9–18 cm^2^ year^−1^) is at the upper limit reported by Nozawa et al. (2021) in their investigation of latitudinal variations in juvenile demography, in line with their findings for a similar latitude (6° S, Karimunjawa, median: 15 cm^2^ year^−1^). But notably, these comparisons do not control for initial colony size which could bias interpretations. The mean growth rates that we observed in massive recruits were higher than those found by Nozawa et al. (2021) in *Porites* juveniles (4.1–7.4 vs. 0.1–5.3 cm^2^ year^−1^), although their study does not include *Porites* colonies within 3° of Peros Banhos’ latitude (and latitude was found to correlate with growth). We observed high mean annual survival rates of 77–93%, which is also at the higher end of the range reported by Nozawa et al. (2021). Studies specifically reporting survival following disturbance have also found similarly high annual survival rates (i.e. exceeding 80%; Gilmour et al. 2013; Morais et al. 2024).

With surveys conducted a year apart we cannot definitively determine the causes of recruit mortality or disappearance. However, examining their last known location provides some useful insight, such as evidence of substrate loss or colony removal (Fig. 4c). It is only possible to speculate on ultimate reasons for the most prevalent cause of mortality—colony disappearance (56–66%; Fig. 4c)—but accidental or deliberate removal by grazers is one plausible driver. Excluding large grazers and corallivores has been found to increase recruit survival in the Chagos Archipelago and elsewhere (McDevitt-Irwin et al. 2024; van der Steeg et al. 2024), supporting its potential influence on recruit mortality here. Additionally, many recruits settled on dead tabular *Acropora* colonies, raising concerns that degradation and crumbling of these coral skeletons could become a common cause of recruit mortality since extensive disintegration can occur within two years of colony death (Morais et al 2022; Sheppard et al. 2002). However, only 10–15% of recruit mortality or disappearance was attributed to substrate loss, suggesting that dead coral generally provided a stable habitat for recruits in the first three years after bleaching here. Further research is needed to assess the influence of ongoing substrate decay on recruit assemblages over longer periods following bleaching since overall reef budgets can become strongly negative for several years and structural collapse can continue to occur up to ten years after bleaching (Engelhardt 2004; Graham et al. 2007; Lange et al. 2023).

### Potential legacy of the marine heatwave

Recruit density was highest the first year after bleaching, whilst both survival *and* growth rates were significantly lower in this first post-bleaching cohort of recruits (2017 recruits) than in the second (2018 recruits; Figs. 2 and S3). Survival and growth rates are likely coupled here—it is well documented that faster growing coral colonies will reach a ‘size-escape threshold’ more quickly, beyond which they have greater survival prospects as a larger colony (Babcock & Mundy 1996; Doropoulos et al. 2012; Randall et al. 2020). This coupling was also observed within morphotaxa, whereby branching colonies (*Acropora* and ‘other branching’) displayed the highest rates of both survival and growth (Fig. 4; Table S10).

Why 2018 recruits achieved higher survival and growth rate than 2017 recruits is harder to ascertain, and cannot be confirmed from the data available in this study. One potential explanation is that the observed lower rates represent a delayed effect of exposure to elevated temperatures. The largest phase of the extended marine heatwave responsible for the 2015/2016 bleaching event in the Indian Ocean occurred between March and June 2016, peaking in May (NOAA Coral Reef Watch; Fig. S2). There are limited data regarding coral spawning times in the Chagos Archipelago, but all records for the central Indian Ocean in the Coral Spawning Database (Baird et al. 2020) report spawning as occurring between October and March (including one record of February spawning in the Chagos Archipelago; Mangubhai et al. 2007; Raj & Edward 2010), suggesting that spawning at Peros Banhos likely occurs within this period. It is unreliable to predict the age of recruits based on their size (since growth can be plastic), but the initial size of recruits in this study (mean: ~ 6 cm^2^; Fig. S1) is similar to sizes reached by 10–15 month-old colonies (Cruz & Harrison 2020; Guest et al. 2014), consistent with if 2017 recruits had settled between February and May 2016. Given the occurrence of the 2016 heatwave shortly after (or even during) the period in which coral spawning likely occurs in the Chagos Archipelago, it is likely that 2017 recruits were exposed to high water temperatures, either indirectly (through their parents or as gametes) or directly (as larvae or recently settled spat). Conversely, since none were visible in April 2017, it is unlikely that 2018 recruits were exposed to the 2016 heatwave.

In addition to causing the well documented bleaching response, exposure to high temperature can reduce gamete size and quality in adult corals (Bouwmeester et al. 2023; Hazraty-Kari et al. 2022), suggesting a potential mechanism for reduced health in 2017 recruits, although smaller gametes do not necessarily lead to lower subsequent survival at later developmental stages (Hazraty-Kari et al. 2022). There is also growing evidence that surviving bleaching events can have delayed negative effects on corals months after temperatures have returned to normal (e.g. by reducing colony growth, physiological condition and reproductive output; Bouwmeester et al. 2023; Cantin & Lough 2014; Gold & Palumbi 2018; Leinbach et al., 2021). Our results provide evidence that heatwaves could also have deleterious legacy effects on the survival and growth of the first colonies to recruit after bleaching. However, further longitudinal monitoring at other reef sites is required to investigate if this trend is observed elsewhere, and more detailed experimental manipulations are required to test associations between early exposure to heat stress and reduced demographic rates later in life.

The lower recruit density observed in 2018 and 2019 (Fig. 2a) could be due to there being fewer reproductive colonies surviving after the bleaching event than before the event—a ‘stock-recruitment effect’ (Gilmour et al. 2013; Hughes et al. 2019). Alternatively, if elevated temperatures indeed reduced coral health and growth (as discussed above), it is possible that the ‘recruits’ observed include some 2016 recruits that settled in the years prior to bleaching but then experienced stunted growth due to the elevated temperatures. Stunted development of such colonies could have led to their misclassification as ‘2017 recruits’, potentially inflating the density of ‘recruits’ reported here (Fig. 2a). Since no images of our quadrats were collected prior to the 2016 bleaching event it is not possible to be certain whether 2017 colonies were present before the bleaching or not. This limitation highlights the benefit of conducting pre-disturbance surveys where possible (e.g. Lachs et al. (2024)). Where this is not feasible—as here—it is still possible to investigate post-disturbance dynamics, but results must be interpreted more cautiously.

### Benthic complexity and recruit vital rates

Greater surface complexity, particularly at centimetre-scales, generally enhances recruitment rates due to there being more spaces providing refuge from grazing and predation (microrefugia), and it is also theorised to facilitate easier settlement by slowing water movement (Abelson & Denny 1997; Graham & Nash 2013; Hamner & Hauri 1981; Hata et al. 2017; Randall et al. 2021; Yanovski & Abelson 2019). Here, however, we observed that within our study areas that were generally flat and featureless in 2017 (i.e. areas dominated by dead tabular Acropora and reef pavement), there were significant negative associations between fractal dimension in 2017 and recruit density and survival in the following years (Fig. 3b).

Within the low complexity areas that this study focused on, slightly higher fine-scale complexity immediately following bleaching-induced mortality resulted in slightly greater recruitment in 2017, but within two years this effect was reversed, and flatter, less complex areas had significantly higher densities of new recruits by 2019. It is possible that microrefugia provision and slowing of water could explain the (non-significant) positive trend in 2017, but a significant negative relationship in 2019 is unexpected. One potential explanation is that the more complex quadrats that had higher recruitment in 2017 subsequently experienced more intense competition between spat during settlement in later years because 2017 recruits had occupied the available microrefugia, ultimately resulting in higher settlement success in areas that were originally marginally less optimal for juvenile coral survival (i.e. in less complex areas (Tilman 1990)).

Colonies in quadrats with higher fine-scale surface complexity (and hence more microrefugia) were less likely to survive their first year following recruitment. This is despite microrefugia’s ability to reduce corallivory (Gallagher & Doropoulos 2017) and indications that corallivory is a significant cause of recruit mortality in the Chagos Archipelago (Fig. 4c; (McDevitt-Irwin et al. 2024)). In theory, the higher densities of recruits observed in 2017 in more complex areas (Fig. 3b) could have caused higher competition between cohorts of recruits in these areas over subsequent years, resulting in greater mortality among recruits due to overgrowth and exclusion. However, we observed competition to be the least frequent cause of mortality (3–4%; Fig. 4c), suggesting that this was not the case here. While we found there to be no significant relationship between fractal dimension and first-year growth, the association had a negative trend with a high level of uncertainty (*p* = 0.056; Table S9), suggesting that the limited range of fractal dimension values in our study may have constrained our ability to detect a significant effect. Future research encompassing a broader range of fractal dimension is needed to more rigorously evaluate if a negative trend also applies between benthic complexity and recruit growth.

High surface complexity following degradation has typically been suggested as an accelerator of reef recovery (Colgan 1987; Guzman & Cortés, 2007) —our results suggest that the relationship between fine-scale complexity and recruitment is not necessarily straightforward nor strictly positive over the first few years of recovery where reefs have been reduced to largely flat, featureless benthos. Longer-term studies that consider multiple metrics of benthic complexity across larger areas are needed to investigate the extent to which the negative relationships we observed are reflected in older communities. Also, our annual photo-survey data are insufficient to determine the ultimate causes of the observed negative relationships and more targeted research into the mechanisms responsible is required to better understand this trend. Surface complexity metrics can easily be extracted from photogrammetry data (e.g. Fukunaga & Burns (2020)) so clarification of a relationship between benthic complexity and recovery dynamics could present a scalable method for identifying patches of reef with high recovery potential over large spatial extents. Surface complexity also influences various aspects of reef ecology, including fish abundance and shoreline protection, so monitoring approaches that quantify structural complexity could also offer valuable contributions to broader ecological assessments (Graham & Nash 2013).

### Tracking recruits using photogrammetry

Given our successful application of photogrammetry here, and the potential offered by new annotation methods, we support prior claims that photogrammetric approaches present a viable and scalable option for long-term monitoring of coral recruits during reef recovery, and can assist in future investigation of post-bleaching recruitment dynamics (Ferrari et al. 2021; Remmers et al. 2024a, b). The logistical challenges of tracking over 1,000 small individuals were overcome by our use of photogrammetry, which allowed the time-consuming tasks of colony measurement and relocation to be conducted ex situ using orthomosaics, enabling inclusion of more colonies than we could have tracked in situ. Whilst easier and faster than doing so in the water, the process of manually annotating orthomosaics was still slow and labour intensive (~ 2 h m^−2^), ultimately limiting us to studying only 12 m^2^ of each 100 m^2^ orthomosaic. However, recent developments in orthomosaic annotation (including AI-assisted automatic and semi-automatic colony segmentation and identification) facilitate much more efficient relocation and measuring of colonies, reducing annotation time by 90% (Pavoni et al. 2022; Remmers et al. 2024a, b). These developments make it plausible to track significantly more individuals in future studies. Additionally, the use of photogrammetry readily provided surface complexity metrics, supplementing the study by enabling investigation of variables that are otherwise challenging to quantify. Whilst photogrammetry can be used to track small features (e.g. coral recruits), the success of this is dependent on the collection of high quality photogrammetry data. Scaled, high-resolution orthomosaics (e.g. under 1 mm pixel^−1^) are necessary to accurately remeasure recruits though time and these require the collection of densely overlapping and high-resolution photographs, paired with the use of Ground Control Points and permanent markers to enable the relocation of individuals (Bayley & Mogg 2020; Lechene et al. 2024).

### Recruit coral cover three years after bleaching

Consideration of changes in coral cover can supplement insights gained from demographic studies (Miller et al. 2016). Total coral cover at Peros Banhos prior to 2015/2016 bleaching was 36% in 2012 (Head et al. 2019). Three years after bleaching, post-bleaching recruits remained relatively small and contributed 2.39% coral cover (Fig. 6). Notably, this value is not *total* cover since it does not include the few large colonies that survived the bleaching (Head et al. 2019). Future research that investigated the demographic rates and coral cover contributions of both new recruits and remnant colonies surviving major disturbances would build on this study and contextualise the role of coral survivors in helping to drive disturbance–recovery dynamics.

One of the fastest recoveries of coral cover documented in scientific literature was at Lighthouse reef in Palau, western Pacific. There, three years after Typhoon Bopha reduced coral cover to zero, coral cover had reached a predicted level of only 5% (Doropoulos et al. 2022). At Peros Banhos, we found that post-bleaching recruits were, as expected, also still considerably short of yielding pre-disturbance levels of coral cover after three years, once again demonstrating that coral reef recovery is a process requiring many years. This was the case even here, at this remote reef that is thought to be one of the least affected by direct human disturbances, under conditions conducive to high recruitment, survival and growth (Fig. 2; Hays et al. 2020; Sheppard et al. 2012). Whilst these appear to be optimal conditions for recovery, it is possible that the archipelago’s isolation may have constrained larval input from external reefs and prevented Peros Banhos from reaching full recovery potential (e.g. Gilmour et al. (2013); although there is evidence that the Chagos Archipelago has surprisingly high larval connectivity within the southeast Indian Ocean (Vogt-Vincent et al. 2024)). Despite a slow initial three years, Lighthouse reef returned to over 60% coral cover by 6 years post-disturbance (Doropoulos et al. 2022), evidencing that recovery of coral cover is not linear and can accelerate rapidly, when given sufficient time and favourable conditions. If reefs are to recover to pre-disturbance states—which they must if they are to continue to provide the ecosystem services for which they are so valued—they must be afforded recovery periods far exceeding three years and likely at least 10 years (Gilmour et al. 2013; Holbrook et al. 2018; Lachs et al. 2024; Sheppard et al. 2008).

Our results reiterate that reef recovery is a slow process, even in a large, remote, no-take MPA with abundant recruits that have fast growth and high survival. Short intervals between climate-induced disturbances will lead to severely altered reef communities, and mitigating the causes of disturbances by delivering international climate change commitments should be a priority. In the meantime, there remains a need to better understand how reef recovery dynamics vary as these processes play an increasingly critical role in shaping future reef health.

## Supplementary Information

Below is the link to the electronic supplementary material.

Supplementary file1 (DOCX 337 KB)

Supplementary file2 (R 37 KB)

Supplementary file3 (CSV 127 KB)

Supplementary file4 (CSV 2 KB)

Supplementary file4 (TXT 2 KB)

## Data Availability

Tables S1 and S2, and Figs. S1–S11 are available in the Online Resources file ESM_1_Supplementary_Information. The data (.csv) and R code used in analyses are also provided within the Online Resources. The data (.csv) and R code used in analyses are provided within the Online Resources.
