# Supplementary material for: Fate-tracking early coral recruits following bleaching in a remote reef ecosystem
Source: Coral Reefs. 2025 Sep 3;44(5):1651–67. doi: 10.1007/s00338-025-02732-8 (PMC12500822; doi:10.1007/s00338-025-02732-8)
Supplement: Supplementary file 1 — Supplementary file1 (DOCX 337 KB) [file 338_2025_2732_MOESM1_ESM.docx]

# Supplementary Information (SI)

**Fate-Tracking Early Coral Recruits Following Bleaching in a Remote Reef Ecosystem**

**Coral Reefs**

**Authors*:*** John E. Stratford ^*1,2,3^, Andrew O.M. Mogg ^4^, Heather J. Koldewey ^5,6^, Liam Lachs^2^, Renata Ferrari^3^, James Guest^2^, Daniel T.I. Bayley ^1^

**Affiliations:**

1. Centre for Biodiversity and Environment Research, University College London, Bloomsbury, London, WC1H 0AG, UK
2. School of Natural and Environmental Sciences, Newcastle University, Newcastle upon Tyne, UK
3. Australian Institute of Marine Science, Townsville, QLD, 4810, Australia
4. Tritonia Scientific Ltd., Dunstaffnage Marine Laboratories, Oban, UK
5. Zoological Society of London, Regent's Park, London, UK
6. Centre for Ecology and Conservation, University of Exeter, Penryn Campus, Cornwall, UK

* corresponding author: j.stratford2@newcastle.ac.uk


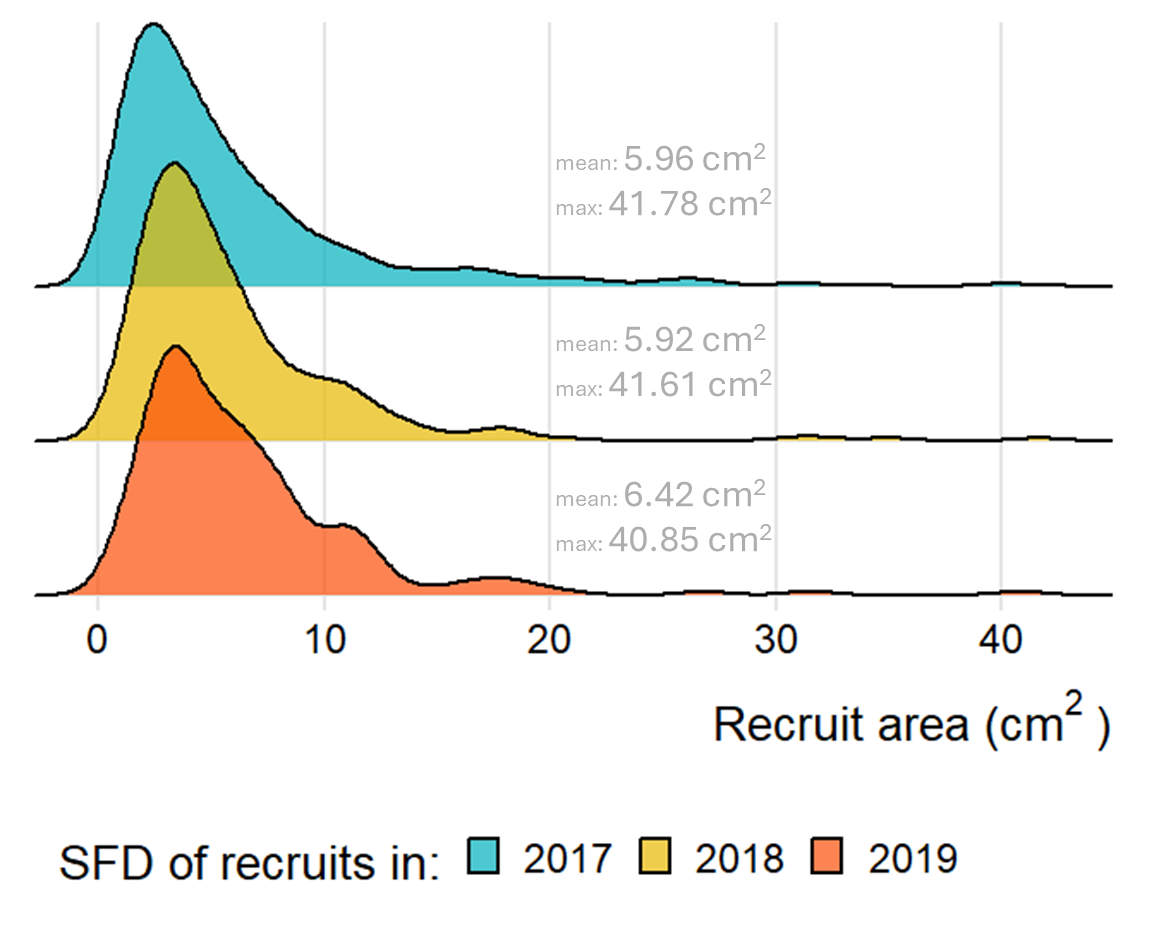


**Figure S1.** Size frequency distribution of all colonies identified as recruits in the year in which they were believed to have been recruited.

**
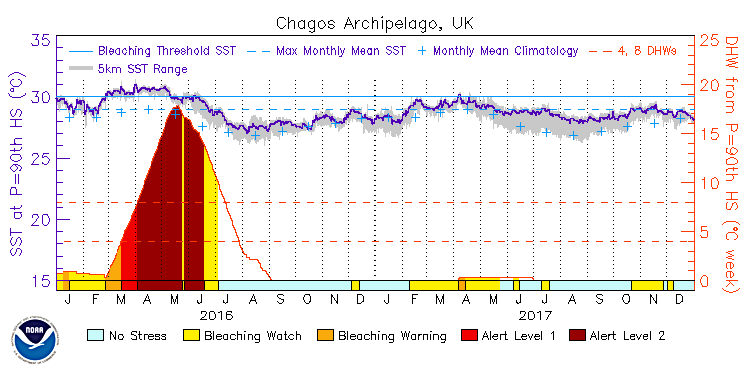
**

**Figure S2.** The Regional Coral Bleaching Heat Stress Gauge for the Chagos Archipelago for 2016 and 2017, accessed from the National Oceanic and Atmospheric Administration’s Coral Reef Watch (<https://coralreefwatch.noaa.gov/product/vs/gauges/chagos_archipelago.php>)

**
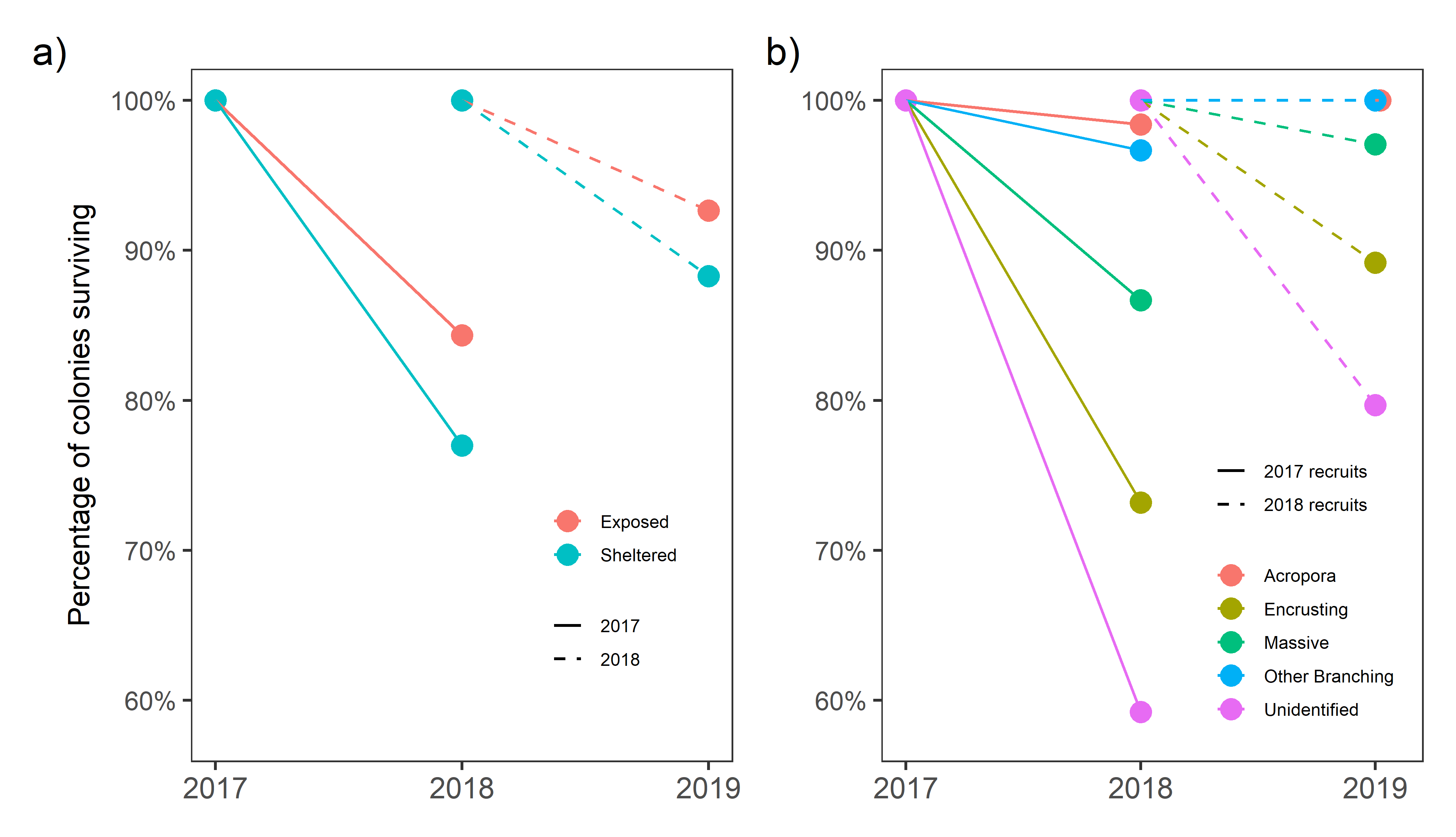
**

**Figure S3.** Survivorship plots of both 2017 and 2018 cohorts of recruits, split by a) exposure and b) morphotaxa. Steeper lines indicate lower first year survival.

**Table S1.** The specifications and settings of **A)** the camera and housing, and **B)** Agisoft Metashape (Agisoft, St. Petersburg, Russia;<https://www.agisoft.com/>) used to produce all 3D models. Table format adapted from Pascoe et al (2021)**.**

| A) Camera and housing specifications and settings | |
| --- | --- |
| Camera | Nikon D750 (24MP) |
| Camera Lens | Nikon Nikkor wide-angle F2.8D AF |
| Dome port | Subal DP-230 9” port |
| Focal length | 20 mm |
| Shutter speed | 1/250 minimum |
| ISO | 100 - 400 |
| Aperture | 4 - 5.6 |
| B) Settings used in 3D model construction in Agisoft Metashape Professional | |
| Software version | Agisoft Metashape Pro v 1.6.3 |
| Align photos | Medium accuracy, generic preselection disabled, infinite key point limit, infinite tie point limit |
| Optimise camera alignment | All default parameters |
| Build Dense Cloud | High quality, Mild depth filtering, Reuse depth maps disabled |
| Build Mesh | Arbitrary surface type, High face count, Interpolation enabled, Calculate vertex colours enabled |
| Build Texture | Generic mapping mode, Mosaic blending mode, Texture size/count 16, 384, Enable hole filling |
| Build Tiled Model | 256 tile size, High face count |
| Build Orthomosaic | Mosaic blending mode, DEM surface, Enable hole filling, Default minimum pixel size (mean = 0.57 (± 0.12) mm pixel^-1^) |
| Build Digital Elevation Model | Point cloud source data, Interpolation enabled |

**Table S2.** The model formulations of all six statistical models used in this study. All statistical analyses were conducted in R v.4.3.2 (R Core Team 2024), using the glmmTMB package (Brooks et al., 2017) to construct GLMMs and the emmeans package (Lenth et al., 2024) to conduct Tukey tests. Model validation was completed using visual assessment of residual plots, aided by the DHARMa R package (Hartig & Lohse, 2022).

| **Model 1:** |
| --- |
| Recruit density ~ Exposure x Recruitment year + Fractal dimension + (1 \| Quadrat), *family = Tweedie (link = log)* |
|  |
| **Model 2:** |
| First-year survival ~ Exposure x Recruitment year + Morphotaxa+ Fractal dimension + Initial recruit size + (1 \| Quadrat), *family = binomial (link = logit)* |
|  |
| **Model 3:** |
| First-year growth ~ Exposure x Recruitment year + Morphotaxa + Fractal dimension + Initial recruit size + (1 \| Quadrat), *family = Gamma (link = log)* |
|  |
| **Model 4:** |
| Recruit density ~ Fractal dimension x Recruitment year + Exposure + (1 \| Quadrat), *family = Tweedie (link = log)* |
|  |
| **Model 5:** |
| First-year survival ~ Morphotaxa x Recruitment year + Exposure + Fractal dimension + Initial recruit size + (1 \| Quadrat), *family = binomial (link = logit)* |
|  |
| **Model 6:** |
| First-year growth ~ Morphotaxa x Exposure x Recruitment year + Fractal dimension + Initial recruit size + (1 \| Quadrat), *family = Gamma (link = log)* |

**Table S3.** Mean recruitment, colony first survival rate and first year growth rate of post-bleaching recruits in the Chagos Archipelago following the 2016 mass bleaching. Growth rate is reported as both planar growth (i.e. the change in colony planar surface area, in cm^2^/yr) and Arithmetic Mean Radius (AMR) growth (i.e. the change in colony radius given colony planar area, assuming the colony is a circle, in cm/yr).

|  |  | Mean quadrat recruitment density | | Colony 1st year survival | | Mean colony 1st year growth | | |
| --- | --- | --- | --- | --- | --- | --- | --- | --- |
| **Recruitment year** | **Exposure** | **Recruit density (recruits/m^2^)** | **n** | **Survival rate** | **n** | **Planar growth rate (cm^2^/yr)** | **AMR growth rate (cm/yr)** | **n** |
| 2017 | Combined | 8.54 | 18 | 0.797 | 595 | 9.16 | 0.80 | 403 |
| 2018 | Combined | 4.05 | 18 | 0.904 | 281 | 11.1 | 0.86 | 214 |
| 2019 | Combined | 2.88 | 18 | NA | NA | NA | NA | NA |
| 2017 | Exposed | 6.1 | 9 | 0.843 | 217 | 7.43 | 0.72 | 161 |
| 2018 | Exposed | 3.83 | 9 | 0.926 | 136 | 9.45 | 0.73 | 110 |
| 2019 | Exposed | 1.78 | 9 | NA | NA | NA | NA | NA |
| 2017 | Sheltered | 11 | 9 | 0.77 | 378 | 10.3 | 0.86 | 242 |
| 2018 | Sheltered | 4.27 | 9 | 0.883 | 145 | 12.9 | 1.00 | 104 |
| 2019 | Sheltered | 3.98 | 9 | NA | NA | NA | NA | NA |

**Table S4.** Colony first survival rate and median/maximum first year growth rate of different morphotaxa of post-bleaching recruits in the Chagos Archipelago following the 2016 mass bleaching. Growth rate is reported as both planar growth (i.e. the change in colony planar surface area, in cm^2^/yr) and Arithmetic Mean Radius (AMR) growth (i.e. the change in colony radius given colony planar area, assuming the colony is a circle, in cm/yr).

|  |  |  | 1st year survival | |  | | 1st year growth | | | |
| --- | --- | --- | --- | --- | --- | --- | --- | --- | --- | --- |
| **Morphotaxa** | **Recruitment year** | **Exposure** | **Survival rate** | **n** | **Median planar growth (cm^2^/yr)** | **Max planar growth (cm^2^/yr)** | | **Median AMR growth (cm/yr)** | **Max AMR growth (cm/yr)** | **n** |
| Acropora | 2017 | Exposed | 1 | 71 | 9.65 | 34 | | 0.90 | 2.27 | 69 |
| Acropora | 2017 | Sheltered | 0.974 | 116 | 10.4 | 39.1 | | 0.91 | 2.19 | 102 |
| Acropora | 2018 | Exposed | 1 | 15 | 13.4 | 34.8 | | 0.90 | 1.86 | 14 |
| Acropora | 2018 | Sheltered | 1 | 21 | 17.8 | 38 | | 1.42 | 2.57 | 20 |
| Encrusting | 2017 | Exposed | 0.742 | 31 | 5.22 | 10.1 | | 0.53 | 0.90 | 14 |
| Encrusting | 2017 | Sheltered | 0.727 | 66 | 6.25 | 26.3 | | 0.80 | 1.80 | 35 |
| Encrusting | 2018 | Exposed | 0.953 | 64 | 4.68 | 97.2 | | 0.60 | 3.01 | 51 |
| Encrusting | 2018 | Sheltered | 0.821 | 56 | 7.41 | 55 | | 0.80 | 2.30 | 35 |
| Massive | 2017 | Exposed | 0.75 | 8 | NA | NA | | NA | NA | 0 |
| Massive | 2017 | Sheltered | 0.892 | 37 | 4.13 | 34.8 | | 0.51 | 1.73 | 24 |
| Massive | 2018 | Exposed | 1 | 9 | 6.63 | 15.1 | | 0.68 | 1.07 | 7 |
| Massive | 2018 | Sheltered | 0.96 | 25 | 7.38 | 19.6 | | 0.76 | 1.43 | 18 |
| Other_Branching | 2017 | Exposed | 0.857 | 14 | 6.76 | 25.7 | | 0.71 | 2.21 | 10 |
| Other_Branching | 2017 | Sheltered | 1 | 46 | 9.6 | 37.8 | | 1.02 | 1.96 | 39 |
| Other_Branching | 2018 | Exposed | 1 | 9 | 20 | 35.6 | | 1.50 | 2.47 | 9 |
| Other_Branching | 2018 | Sheltered | 1 | 18 | 15 | 47.8 | | 1.10 | 2.34 | 16 |
| Unidentified | 2017 | Exposed | 0.763 | 93 | 4.4 | 17.6 | | 0.53 | 1.43 | 68 |
| Unidentified | 2017 | Sheltered | 0.451 | 113 | 7.25 | 17.1 | | 0.78 | 1.63 | 42 |
| Unidentified | 2018 | Exposed | 0.821 | 39 | 2.86 | 15.6 | | 0.37 | 1.03 | 29 |
| Unidentified | 2018 | Sheltered | 0.76 | 25 | 7.97 | 17.6 | | 0.84 | 1.38 | 15 |

| **Summary – Model 1, Recruitment** | | | | |  |
| --- | --- | --- | --- | --- | --- |
|  | **Estimate** | **Std. Error** | **z value** | **Pr(>\|z\|)** |  |
| (Intercept) | 12.2892 | 8.2251 | 1.494 | 0.1351 |  |
| ExposureSheltered | 0.8719 | 0.3428 | 2.543 | 0.011 |  |
| Year2018 | -0.3625 | 0.3124 | -1.16 | 0.246 |  |
| Year2019 | -1.0805 | 0.3461 | -3.122 | 0.0018 |  |
| Initial_fd | -5.0363 | 3.8738 | -1.3 | 0.1936 |  |
| ExposureSheltered:Year2018 | -0.6287 | 0.4216 | -1.491 | 0.1359 |  |
| ExposureSheltered:Year2019 | -0.1013 | 0.4631 | -0.219 | 0.8268 |  |
| **Emmeans contrasts (ignoring exposure) – Model 1, Recruitment** | | | |  |  |
| **contrast** | **estimate** | **SE** | **df** | **z.ratio** | **p.value** |
| Year2017 - Year2018 | 0.677 | 0.211 | Inf | 3.213 | 0.0038 |
| Year2017 - Year2019 | 1.131 | 0.23 | Inf | 4.928 | <.0001 |
| Year2018 - Year2019 | 0.454 | 0.239 | Inf | 1.904 | 0.1374 |
| **Emmeans contrasts (considering exposure) – Model 1, Recruitment** | | | |  |  |
| **contrast** | **estimate** | **SE** | **df** | **z.ratio** | **p.value** |
| Exposed Year2017 - Sheltered Year2017 | -0.8719 | 0.343 | Inf | -2.543 | 0.1117 |
| Exposed Year2017 - Exposed Year2018 | 0.3625 | 0.312 | Inf | 1.16 | 0.8557 |
| Exposed Year2017 - Sheltered Year2018 | 0.1193 | 0.363 | Inf | 0.329 | 0.9995 |
| Exposed Year2017 - Exposed Year2019 | 1.0805 | 0.346 | Inf | 3.122 | 0.0222 |
| Exposed Year2017 - Sheltered Year2019 | 0.3099 | 0.368 | Inf | 0.841 | 0.9598 |
| Sheltered Year2017 - Exposed Year2018 | 1.2344 | 0.347 | Inf | 3.562 | 0.005 |
| Sheltered Year2017 - Sheltered Year2018 | 0.9912 | 0.283 | Inf | 3.504 | 0.0061 |
| Sheltered Year2017 - Exposed Year2019 | 1.9523 | 0.373 | Inf | 5.238 | <.0001 |
| Sheltered Year2017 - Sheltered Year2019 | 1.1818 | 0.305 | Inf | 3.879 | 0.0015 |
| Exposed Year2018 - Sheltered Year2018 | -0.2432 | 0.366 | Inf | -0.664 | 0.9858 |
| Exposed Year2018 - Exposed Year2019 | 0.718 | 0.351 | Inf | 2.043 | 0.318 |
| Exposed Year2018 - Sheltered Year2019 | -0.0526 | 0.374 | Inf | -0.141 | 1 |
| Sheltered Year2018 - Exposed Year2019 | 0.9612 | 0.391 | Inf | 2.457 | 0.1371 |
| Sheltered Year2018 - Sheltered Year2019 | 0.1906 | 0.323 | Inf | 0.59 | 0.9917 |
| Exposed Year2019 - Sheltered Year2019 | -0.7706 | 0.399 | Inf | -1.933 | 0.3821 |

**Table S5.** The model summary of Model 1 (as returned by glmmTMB) and the results of emmeans contrasts from Model 1, investigating pairwise relationships between recruitment and exposure, and the number of years since bleaching.

**Table S6.** The model summary of Model 4 (as returned by glmmTMB) and the results of emtrends from Model 4, investigating the relationship between recruitment and fractal dimension in 2017, in each of the years since bleaching.

| **Summary – Model 4** **, Recruitment** | | | | |  |
| --- | --- | --- | --- | --- | --- |
|  | Estimate | Std. Error | z value | Pr(>\|z\|) |  |
| (Intercept) | -8.9959 | 8.8117 | -1.021 | 0.3073 |  |
| Initial_fd | 5.0108 | 4.1489 | 1.208 | 0.22715 |  |
| Year2018 | 23.1707 | 8.3889 | 2.762 | 0.00574 |  |
| Year2019 | 64.0712 | 11.2245 | 5.708 | 1.14E-08 |  |
| ExposureSheltered | 0.5908 | 0.2671 | 2.212 | 0.02699 |  |
| Initial_fd:Year2018 | -11.2269 | 3.943 | -2.847 | 0.00441 |  |
| Initial_fd:Year2019 | -30.7446 | 5.3138 | -5.786 | 7.22E-09 |  |
| **Emtrends – Model 4** **, Recruitment** | | | | |  |
| **Year** | **Initial_fd.trend** | **SE** | **df** | **z.ratio** | **p.value** |
| 2017 | 5.01 | 4.15 | Inf | 1.208 | 0.2271 |
| 2018 | -6.22 | 4.82 | Inf | -1.291 | 0.1968 |
| 2019 | -25.73 | 5.91 | Inf | -4.353 | <.0001 |

| **Summary – Model 2, Survival** |  |  |  |  |  |
| --- | --- | --- | --- | --- | --- |
|  | **Estimate** | **Std. Error** | **z value** | **Pr(>\|z\|)** |  |
| (Intercept) | 28.51347 | 11.04379 | 2.582 | 0.00983 |  |
| ExposureSheltered | -0.6264 | 0.40251 | -1.556 | 0.11965 |  |
| Recruitment_year2018 | 0.84798 | 0.42295 | 2.005 | 0.04497 |  |
| MorphotaxaEncrusting | -3.07908 | 0.63821 | -4.825 | 1.40E-06 |  |
| MorphotaxaMassive | -1.23708 | 0.85123 | -1.453 | 0.14615 |  |
| MorphotaxaOther_Branching | 0.03494 | 1.17421 | 0.03 | 0.97626 |  |
| MorphotaxaUnidentified | -3.96546 | 0.62517 | -6.343 | 2.25E-10 |  |
| Initial_fd | -11.3672 | 5.17484 | -2.197 | 0.02805 |  |
| Initial_area | 0.06483 | 0.03275 | 1.98 | 0.04772 |  |
| ExposureSheltered:Recruitment_year2018 | -0.13424 | 0.52976 | -0.253 | 0.79995 |  |
| **Emmeans contrasts (ignoring exposure) – Model 2, Survival** | | |  |  |  |
| **contrast** | **estimate** | **SE** | **df** | **z.ratio** | **p.value** |
| Recruitment_year2017 - Recruitment_year2018 | -0.781 | 0.274 | Inf | -2.854 | 0.0043 |
| **Emmeans contrasts (considering exposure) – Model 2, Survival** | | |  |  |  |
| **contrast** | **estimate** | **SE** | **df** | **z.ratio** | **p.value** |
| Exposed Recruitment_year2017 - Sheltered Recruitment_year2017 | 0.6264 | 0.403 | Inf | 1.556 | 0.4039 |
| Exposed Recruitment_year2017 - Exposed Recruitment_year2018 | -0.848 | 0.423 | Inf | -2.005 | 0.1861 |
| Exposed Recruitment_year2017 - Sheltered Recruitment_year2018 | -0.0873 | 0.463 | Inf | -0.188 | 0.9976 |
| Sheltered Recruitment_year2017 - Exposed Recruitment_year2018 | -1.4744 | 0.46 | Inf | -3.205 | 0.0074 |
| Sheltered Recruitment_year2017 - Sheltered Recruitment_year2018 | -0.7137 | 0.333 | Inf | -2.141 | 0.1403 |
| Exposed Recruitment_year2018 - Sheltered Recruitment_year2018 | 0.7606 | 0.505 | Inf | 1.506 | 0.4336 |

**Table S7.** The model summary of Model 2 (as returned by glmmTMB) and the results of emmeans contrasts from Model 2, investigating pairwise relationships between colony first year survival and exposure, and the number of years since bleaching.

| **Summary – Model 5, Survival by Morphotaxa** |  |  |  |  |  |
| --- | --- | --- | --- | --- | --- |
|  | **Estimate** | **Std. Error** | **z value** | **Pr(>\|z\|)** |  |
| (Intercept) | 2.84E+01 | 1.10E+01 | 2.58 | 0.00987 |  |
| Recruitment_year2018 | 1.24E+01 | 6.13E+02 | 0.02 | 0.98391 |  |
| MorphotaxaEncrusting | -2.99E+00 | 6.56E-01 | -4.562 | 5.07E-06 |  |
| MorphotaxaMassive | -1.09E+00 | 9.54E-01 | -1.139 | 0.25469 |  |
| MorphotaxaOther_Branching | -5.18E-02 | 1.18E+00 | -0.044 | 0.96494 |  |
| MorphotaxaUnidentified | -3.86E+00 | 6.29E-01 | -6.143 | 8.12E-10 |  |
| ExposureSheltered | -6.73E-01 | 3.59E-01 | -1.873 | 0.06109 |  |
| Initial_fd | -1.13E+01 | 5.15E+00 | -2.2 | 0.02782 |  |
| Initial_area | 6.48E-02 | 3.27E-02 | 1.982 | 0.04753 |  |
| Recruitment_year2018:MorphotaxaEncrusting | -1.16E+01 | 6.13E+02 | -0.019 | 0.98492 |  |
| Recruitment_year2018:MorphotaxaMassive | -1.18E+01 | 6.13E+02 | -0.019 | 0.98468 |  |
| Recruitment_year2018:MorphotaxaOther_Branching | 1.39E+01 | 7.66E+05 | 0 | 0.99999 |  |
| Recruitment_year2018:MorphotaxaUnidentified | -1.17E+01 | 6.13E+02 | -0.019 | 0.98484 |  |
| **Emmeans contrasts: 2017 recruits vs 2018 recruits – Model 5** | | | | |  |
| **Morphotaxa** | **estimate** | **SE** | **df** | **z.ratio** | **p.value** |
| Acropora | -12.364 | 613.2 | Inf | -0.02 | 0.9839 |
| Encrusting | -0.774 | 0.4 | Inf | -1.882 | 0.0598 |
| Massive | -0.588 | 1.3 | Inf | -0.462 | 0.6438 |
| Other_Branching | -26.235 | 765493.6 | Inf | 0 | 1 |
| Unidentified | -0.717 | 0.4 | Inf | -1.97 | 0.0489 |
| **Emmeans contrasts: annual survival between morphotaxa – Model 5** | | | |  |  |
| **Recruitment_year = 2017:** | | |  |  |  |
| **contrast** | **estimate** | **SE** | **df** | **z.ratio** | **p.value** |
| Acropora - Encrusting | 2.9906 | 0.7 | Inf | 4.562 | <.0001 |
| Acropora - Massive | 1.0871 | 1 | Inf | 1.139 | 0.7858 |
| Acropora - Other_Branching | 0.0518 | 1.2 | Inf | 0.044 | 1 |
| Acropora - Unidentified | 3.8632 | 0.6 | Inf | 6.143 | <.0001 |
| Encrusting - Massive | -1.9035 | 0.8 | Inf | -2.403 | 0.1146 |
| Encrusting - Other_Branching | -2.9388 | 1.1 | Inf | -2.782 | 0.043 |
| Encrusting - Unidentified | 0.8726 | 0.3 | Inf | 2.717 | 0.0515 |
| Massive - Other_Branching | -1.0353 | 1.3 | Inf | -0.819 | 0.9248 |
| Massive - Unidentified | 2.7761 | 0.8 | Inf | 3.62 | 0.0027 |
| Other_Branching - Unidentified | 3.8113 | 1 | Inf | 3.676 | 0.0022 |
|  |  |  |  |  |  |
| **Recruitment_year = 2018:** | | |  |  |  |
| **contrast** | **estimate** | **SE** | **df** | **z.ratio** | **p.value** |
| Acropora - Encrusting | 14.581 | 613.2 | Inf | 0.024 | 1 |
| Acropora - Massive | 12.8634 | 613.2 | Inf | 0.021 | 1 |
| Acropora - Other_Branching | -13.8191 | 765493.8 | Inf | 0 | 1 |
| Acropora - Unidentified | 15.5105 | 613.2 | Inf | 0.025 | 1 |
| Encrusting - Massive | -1.7175 | 1.1 | Inf | -1.594 | 0.501 |
| Encrusting - Other_Branching | -28.4001 | 765493.6 | Inf | 0 | 1 |
| Encrusting - Unidentified | 0.9296 | 0.5 | Inf | 2.013 | 0.2595 |
| Massive - Other_Branching | -26.6825 | 765493.6 | Inf | 0 | 1 |
| Massive - Unidentified | 2.6471 | 1.1 | Inf | 2.432 | 0.1068 |
| Other_Branching - Unidentified | 29.3296 | 765493.6 | Inf | 0 | 1 |

**Table S8.** The model summary of Model 5 (as returned by glmmTMB) and the results of emmeans contrasts from Model 5, investigating pairwise relationships between first year survival of 2017 recruits against 2018 recruits for each morphotaxa and between first year survival of different morphotaxa in both 2017 and 2018.

**Table S9.** The model summary of Model 3 (as returned by glmmTMB) and the results of emmeans contrasts from Model 3, investigating pairwise relationships between first year growth and exposure, and the number of years since bleaching.

| **Summary – Model 3, Growth** |  |  |  |  |  |
| --- | --- | --- | --- | --- | --- |
|  | **Estimate** | **Std. Error** | **z value** | **Pr(>\|z\|)** |  |
| (Intercept) | 5.951408 | 2.082166 | 2.858 | 0.00426 |  |
| ExposureSheltered | 0.213807 | 0.081429 | 2.626 | 0.00865 |  |
| Recruitment_year2018 | 0.115115 | 0.104585 | 1.101 | 0.27103 |  |
| MorphotaxaEncrusting | -0.42146 | 0.092692 | -4.547 | 5.44E-06 |  |
| MorphotaxaMassive | -0.68866 | 0.126864 | -5.428 | 5.69E-08 |  |
| MorphotaxaOther_Branching | 0.008988 | 0.106139 | 0.085 | 0.93251 |  |
| MorphotaxaUnidentified | -0.63006 | 0.084541 | -7.453 | 9.15E-14 |  |
| Initial_fd | -1.86291 | 0.976416 | -1.908 | 0.0564 |  |
| Initial_area | 0.04753 | 0.005508 | 8.629 | < 2e-16 |  |
| ExposureSheltered:Recruitment_year2018 | 0.207235 | 0.133586 | 1.551 | 0.12082 |  |
| **Emmeans contrasts (ignoring exposure) - Model 3, Growth** | | |  |  |  |
| **contrast** | **estimate** | **SE** | **df** | **z.ratio** | **p.value** |
| Recruitment_year2017 - Recruitment_year2018 | -0.219 | 0.0722 | Inf | -3.03 | 0.0024 |
| **Emmeans contrasts (considering exposure) - Model 3, Growth** | | |  |  |  |
| **contrast** | **estimate** | **SE** | **df** | **z.ratio** | **p.value** |
| Exposed Recruitment_year2017 - Sheltered Recruitment_year2017 | -0.2138 | 0.0814 | Inf | -2.626 | 0.0429 |
| Exposed Recruitment_year2017 - Exposed Recruitment_year2018 | -0.1151 | 0.1046 | Inf | -1.101 | 0.6891 |
| Exposed Recruitment_year2017 - Sheltered Recruitment_year2018 | -0.5362 | 0.1039 | Inf | -5.162 | <.0001 |
| Sheltered Recruitment_year2017 - Exposed Recruitment_year2018 | 0.0987 | 0.0932 | Inf | 1.059 | 0.7145 |
| Sheltered Recruitment_year2017 - Sheltered Recruitment_year2018 | -0.3224 | 0.0917 | Inf | -3.515 | 0.0025 |
| Exposed Recruitment_year2018 - Sheltered Recruitment_year2018 | -0.421 | 0.1065 | Inf | -3.954 | 0.0004 |

| **Summary – Model 6, Growth by Morphotaxa** | | | | |  |
| --- | --- | --- | --- | --- | --- |
|  | **Estimate** | **Std. Error** | **z value** | **Pr(>\|z\|)** |  |
| (Intercept) | 5.540639 | 2.098307 | 2.641 | 0.008278 |  |
| Encrusting | -0.79545 | 0.220254 | -3.612 | 0.000304 |  |
| Massive | -0.52282 | 0.458192 | -1.141 | 0.253851 |  |
| Other_Branching | -0.16401 | 0.253989 | -0.646 | 0.518451 |  |
| Unidentified | -0.78597 | 0.128411 | -6.121 | 9.32E-10 |  |
| ExposureSheltered | -0.00446 | 0.118923 | -0.038 | 0.970085 |  |
| 2018 | 0.03533 | 0.221701 | 0.159 | 0.873386 |  |
| Initial_fd | -1.6258 | 0.985759 | -1.649 | 0.09909 |  |
| Initial_area | 0.049696 | 0.005538 | 8.974 | < 2e-16 |  |
| Encrusting:ExposureSheltered | 0.600704 | 0.265678 | 2.261 | 0.023758 |  |
| Massive:ExposureSheltered | -0.12294 | 0.424794 | -0.289 | 0.772274 |  |
| Other_Branching:ExposureSheltered | 0.137084 | 0.290542 | 0.472 | 0.637054 |  |
| Unidentified:ExposureSheltered | 0.530714 | 0.189542 | 2.8 | 0.00511 |  |
| Encrusting:2018 | 0.371581 | 0.316063 | 1.176 | 0.239733 |  |
| Massive:2018 | -0.08465 | 0.298791 | -0.283 | 0.776947 |  |
| Other_Branching:2018 | 0.716034 | 0.409091 | 1.75 | 0.080066 |  |
| Unidentified:2018 | -0.26779 | 0.27663 | -0.968 | 0.333017 |  |
| ExposureSheltered:2018 | 0.478196 | 0.290454 | 1.646 | 0.099687 |  |
| Encrusting:ExposureSheltered:2018 | -0.74913 | 0.413056 | -1.814 | 0.069735 |  |
| Massive:ExposureSheltered:2018 | NA | NA | NA | NA |  |
| Other_Branching:ExposureSheltered:2018 | -0.83447 | 0.502021 | -1.662 | 0.096468 |  |
| Unidentified:ExposureSheltered:2018 | -0.12319 | 0.403761 | -0.305 | 0.76028 |  |
| **Emmeans contrasts (averaged over year and exposure) – Model 6, growth between morphotaxa** | | | | |  |
| **contrast** | **estimate** | **SE** | **df** | **z.ratio** | **p.value** |
| Acropora - Encrusting | 0.4966 | 0.103 | Inf | 4.81 | <.0001 |
| Acropora - Massive | nonEst | NA | NA | NA | NA |
| Acropora - Other_Branching | -0.0539 | 0.126 | Inf | -0.43 | 0.9734 |
| Acropora - Unidentified | 0.6853 | 0.103 | Inf | 6.644 | <.0001 |
| Encrusting - Massive | nonEst | NA | NA | NA | NA |
| Encrusting - Other_Branching | -0.5505 | 0.126 | Inf | -4.372 | 0.0001 |
| Encrusting - Unidentified | 0.1887 | 0.102 | Inf | 1.859 | 0.2461 |
| Massive - Other_Branching | nonEst | NA | NA | NA | NA |
| Massive - Unidentified | nonEst | NA | NA | NA | NA |
| Other_Branching - Unidentified | 0.7392 | 0.125 | Inf | 5.911 | <.0001 |

**Table S10.** The model summary of Model 6 (as returned by glmmTMB) and the results of emmeans contrasts from Model 6, investigating pairwise relationships between first year growth and different morphotaxa.

**Table S11.** The results of emmeans contrasts from Model 6, investigating pairwise relationships between first year growth in 2017 recruits and 2018 recruits for each morphotaxa.

| **Emmeans contrasts – Model 6, Morphotaxa growth by year and exposure** | | | | | |
| --- | --- | --- | --- | --- | --- |
| **contrast** | **estimate** | **SE** | **df** | **z.ratio** | **p.value** |
| **Acropora:** | |  |  |  |  |
| Exposed2017-Sheltered2017 | 0.00446 | 0.119 | Inf | 0.038 | 1 |
| Exposed2017-Exposed2018 | -0.03533 | 0.222 | Inf | -0.159 | 0.9986 |
| Exposed2017-Sheltered2018 | -0.50907 | 0.194 | Inf | -2.623 | 0.0432 |
| Sheltered2017-Exposed2018 | -0.03979 | 0.215 | Inf | -0.185 | 0.9977 |
| Sheltered2017-Sheltered2018 | -0.51353 | 0.185 | Inf | -2.769 | 0.0288 |
| Exposed2018-Sheltered2018 | -0.47374 | 0.265 | Inf | -1.786 | 0.2801 |
| **Encrusting:** |  |  |  |  |  |
| Exposed2017-Sheltered2017 | -0.59624 | 0.238 | Inf | -2.503 | 0.0594 |
| Exposed2017-Exposed2018 | -0.40691 | 0.227 | Inf | -1.795 | 0.2756 |
| Exposed2017-Sheltered2018 | -0.73222 | 0.238 | Inf | -3.081 | 0.0111 |
| Sheltered2017-Exposed2018 | 0.18933 | 0.167 | Inf | 1.135 | 0.6675 |
| Sheltered2017-Sheltered2018 | -0.13598 | 0.182 | Inf | -0.746 | 0.8785 |
| Exposed2018-Sheltered2018 | -0.32531 | 0.165 | Inf | -1.973 | 0.1982 |
| **Massive:** |  |  |  |  |  |
| Exposed2017-Sheltered2017 | nonEst | NA | NA | NA | NA |
| Exposed2017-Exposed2018 | nonEst | NA | NA | NA | NA |
| Exposed2017-Sheltered2018 | nonEst | NA | NA | NA | NA |
| Sheltered2017-Exposed2018 | -0.07808 | 0.323 | Inf | -0.241 | 0.9684 |
| Sheltered2017-Sheltered2018 | -0.42888 | 0.234 | Inf | -1.832 | 0.1591 |
| Exposed2018-Sheltered2018 | -0.3508 | 0.335 | Inf | -1.047 | 0.5474 |
| **Other_Branching:** | |  |  |  |  |
| Exposed2017-Sheltered2017 | -0.13262 | 0.267 | Inf | -0.497 | 0.9598 |
| Exposed2017-Exposed2018 | -0.75136 | 0.345 | Inf | -2.175 | 0.1302 |
| Exposed2017-Sheltered2018 | -0.52771 | 0.303 | Inf | -1.741 | 0.3026 |
| Sheltered2017-Exposed2018 | -0.61874 | 0.279 | Inf | -2.22 | 0.1178 |
| Sheltered2017-Sheltered2018 | -0.39509 | 0.223 | Inf | -1.77 | 0.2879 |
| Exposed2018-Sheltered2018 | 0.22365 | 0.313 | Inf | 0.715 | 0.8913 |
| **Unidentified:** | |  |  |  |  |
| Exposed2017-Sheltered2017 | -0.52625 | 0.147 | Inf | -3.572 | 0.002 |
| Exposed2017-Exposed2018 | 0.23246 | 0.167 | Inf | 1.396 | 0.5019 |
| Exposed2017-Sheltered2018 | -0.6488 | 0.214 | Inf | -3.028 | 0.0131 |
| Sheltered2017-Exposed2018 | 0.75872 | 0.181 | Inf | 4.186 | 0.0002 |
| Sheltered2017-Sheltered2018 | -0.12254 | 0.226 | Inf | -0.543 | 0.9485 |
| Exposed2018-Sheltered2018 | -0.88126 | 0.239 | Inf | -3.692 | 0.0013 |

**Bibliography:**

Brooks, M. E., Kristensen, K., Benthem, K. J. van, Magnusson, A., Berg, C. W., Nielsen, A., Skaug, H. J., Mächler, M., & Bolker, B. M. (2017). glmmTMB Balances Speed and Flexibility Among Packages for Zero-inflated Generalized Linear Mixed Modeling. *The R Journal*, *9*(2), 378–400. https://doi.org/10.3929/ethz-b-000240890

Hartig, F., & Lohse, L. (2022). *DHARMa: Residual Diagnostics for Hierarchical (Multi-Level / Mixed) Regression Models* (Version 0.4.6) [Computer software]. https://cran.r-project.org/web/packages/DHARMa/index.html

Lenth, R. V., Bolker, B., Buerkner, P., Giné-Vázquez, I., Herve, M., Jung, M., Love, J., Miguez, F., Piaskowski, J., Riebl, H., & Singmann, H. (2024). *emmeans: Estimated Marginal Means, aka Least-Squares Means* (Version 1.10.3) [Computer software]. https://cran.r-project.org/web/packages/emmeans/index.html

Pascoe, K. H., Fukunaga, A., Kosaki, R. K., & Burns, J. H. R. (2021). 3D assessment of a coral reef at Lalo Atoll reveals varying responses of habitat metrics following a catastrophic hurricane. *Scientific Reports*, *11*(1), Article 1. https://doi.org/10.1038/s41598-021-91509-4
